# Socio-cognitive constraints and opportunities for sustainable intensification in South Asia: insights from fuzzy cognitive mapping in coastal Bangladesh

**DOI:** 10.1007/s10668-021-01342-y

**Published:** 2021-04-03

**Authors:** Sreejith Aravindakshan, Timothy J. Krupnik, Sumona Shahrin, Pablo Tittonell, Kadambot H. M. Siddique, Lenora Ditzler, Jeroen C. J. Groot

**Affiliations:** 1 Farming Systems Ecology Group, Wageningen University & Research, P.O. Box 430, 6700 AK Wageningen, The Netherlands; 2 International Maize and Wheat Improvement Center (CIMMYT), House 10/B, Road 53, Gulshan-2, Dhaka, 1213 Bangladesh; 3 Instituto Nacional de Tecnología Agropecuaria (INTA), 1439-1033, 1033 Rivadavia, Buenos Aires Argentina; 4 Groningen Institute of Evolutionary Life Sciences, Groningen University, Groningen, The Netherlands; 5 Bioversity International, Viale dei Tre Denari, 472/a, 00054 Maccarese (Fiumicino), Italy; 6 International Maize and Wheat Improvement Center (CIMMYT), Carretera México-Veracruz, Km. 45, El Batán, 56237 Texcoco, México; 7 The UWA Institute of Agriculture, The University of Western Australia, Perth, WA 6001 Australia

**Keywords:** Socio-cognitive model, Systems analysis, Sustainable intensification, Semi-quantitative approach, Winding stairs

## Abstract

Appreciating and dealing with the plurality of farmers’ perceptions and their contextual knowledge and perspectives of the functioning and performance of their agroecosystems—in other words, their ‘mental models’—is central for appropriate and sustainable agricultural development. In this respect, the sustainable development goals (SDGs) aim to eradicate poverty and food insecurity by 2030 by envisioning social inclusivity that incorporates the preferences and knowledge of key stakeholders, including farmers. Agricultural development interventions and policies directed at sustainable intensification (SI), however, do not sufficiently account for farmers’ perceptions, beliefs, priorities, or interests. Considering two contrasting agroecological systems in coastal Bangladesh, we used a fuzzy cognitive mapping (FCM)-based simulation and sensitivity analysis of mental models of respondents of different farm types from 240 farm households. The employed FCM mental models were able to (1) capture farmers’ perception of farming system concepts and relationships for each farm type and (2) assess the impact of external interventions (drivers) on cropping intensification and food security. We decomposed the FCM models’ variance into the first-order sensitivity index (SVI) and total sensitivity index (TSI) using a winding stairs algorithm. Both within and outside polder areas, the highest TSIs (35–68%) were observed for effects of agricultural extension on changes in other concepts in the map, particularly food security and income (SI indicators), indicating the importance of extension programs for SI. Outside polders, drainage and micro-credit were also influential; within polders, the availability of micro-credit appears to affect farmer perceptions of SI indicators more than drainage. This study demonstrated the importance of reflection on the differing perspectives of farmers both within and outside polders to identify entry points for development interventions. In addition, the study underscores the need for micro-farming systems-level research to assess the context-based feasibility of introduced interventions as perceived by farmers of different farm types.

**Supplementary Information:**

The online version contains supplementary material available at 10.1007/s10668-021-01342-y.

## Introduction

Achieving the sustainable development goals (SDGs) is crucial for ending poverty and food insecurity in developing countries. The aim is to achieve these outcomes by 2030 by achieving social inclusivity that addresses the preferences and knowledge of key stakeholders in the development process (Gupta & Vegelin, 2016). Agricultural development in South Asia has typically focused on improved crop varieties and the extension of novel agronomic technologies, with much less attention to the perceptions, beliefs, and priorities of farmers or the ways that these influence their decisions to change or improve farm and crop husbandry practices (Chaudhuri et al., 2020). Sustainable intensification (SI)—an approach aimed at increasing agricultural productivity while reducing environmental and social trade-offs in agricultural development—has become increasingly important in SDGs (Firbank et al., 2018; Rockström et al., 2017). SI is also important for the climate-risk-prone and impoverished coastal zones of South Asia (Aryal et al., 2019; Emran et al., 2019), including coastal areas in India and southern Bangladesh, where approximately 400 million vulnerable people derive their livelihoods, primarily in rural areas (Aravindakshan et al., 2020). Accomplishing the challenging goals associated with SI and the SGDs are particularly relevant for the region’s coastal zones, where millions of farmers compete for land and water resources while dealing with constant challenges, including waterlogging, soil and water salinity, cyclones, extreme weather events, rising sea levels, and poor infrastructural and market development (Akter & Ahmed, 2020; Akter et al., 2016).

In the last half-century, coastal embankments or dykes known as polders have been constructed in Bangladesh to control oceanic intrusion and prevent excessive waterlogging (World Bank, 1990). The lack of maintenance and canal obstructions and diversions from farmers competing for resources—for example, those who require freshwater for irrigating crops and prawn/shrimp producers channeling brackish water—have rendered many protective and water control structures dysfunctional, with increased siltation of canals (Aravindakshan et al., 2020; Kabir et al., 2016). Although primarily intended for flood control, the construction of polders has transformed how agricultural water management functions during the cool, dry winter ‘*rabi*’ season (from November to April). Farmers within polders experience water scarcity and post-monsoon season drainage issues during the *rabi* season due to land subsidence, as well as problems with the control and maintenance of sluice gates. Farmers located just north and outside the polders also experience tidal water inundation in the wet season. Both within and outside polders, almost two million households are fallowing their land during the *rabi* season due to fresh water scarcity and to avert risks in the absence of context-appropriate policies that address the interests and priorities of farmers, which contribute in part to food insecurity and subsistence below the poverty line (Aravindakshan et al., 2020; Krupnik et al., 2017).

Approximately USD 500 million in funds has been requested by the Government of Bangladesh (GoB) from foreign development donors to develop surface water irrigation resources to transition farmers from monsoon season rice followed by the dry *rabi* season land fallowing to intensified double cropping in coastal Bangladesh. These goals align roughly with the SI objectives, which encourage multiple cropping within the same field within a single calendar year (Krupnik et al., 2017; Pretty & Bharucha, 2014). Policy emphasis has, however, been to increase dry season monocropping of irrigated ‘*boro*’ rice during the winter dry season in this region (MOA and FAO 2013), largely to ensure food security, as rice is Bangladesh’s primary staple. It also aims to achieve the strategic goal of shifting *boro* rice entirely or partially to the southern coasts to offset the increasing energy costs and massive depletion of groundwater as a result of intensive *boro* production in northern Bangladesh (Qureshi et al., 2015). The feasibility of this approach is questionable, *in lieu* of the farmers’ preferences to abandon agriculture or grow low-yielding but low-input-dependent pulses in the dry season (Aravindakshan et al., 2020; Schulthess et al., 2019). In addition, the region experiences increasing soil and water salinity as the dry season progresses—a situation exacerbated by water competition as described above—and extreme weather events, and is at risk of climate change-induced sea-level rise (Krupnik et al., 2017; Qureshi et al., 2015).

Rather than promoting crops and farming practices that may not be compatible with the beliefs, priorities, and aspirations of rural communities in coastal areas, approaches that aim for contextually appropriate innovations could assist in reaching SI in these marginal yet densely populated environments. The perceived understanding of farming systems may also differ from farmer to farmer or among different types of farmers, as a result of the heterogeneity in socioeconomic and biophysical circumstances that affect rural communities. Differences in decision-making frameworks among farmers belonging to different farm types also remain poorly understood.^1^

In the coastal areas of Bangladesh, few studies have systematically approached these issues or developed an understanding of how farmers conceptualize the constraints and opportunities associated with their farming systems. We used fuzzy cognitive mapping (FCM) to study the socio-cognitive systems of farmers belonging to different farm types both within and outside polder areas. Our goal was to identify gaps and overlaps in farmers’ understanding of their farming systems and development priorities aimed at using surface water irrigation to encourage double cropping and intensification in the dry season. The main objective of these FCM models was to explore the understanding of farmers in different situations (within and outside polders) of the factors/drivers and processes that affect the functioning of their farms and their decision- making, which in turn could affect sustainable agricultural intensification. Although context-specific and focused on coastal Bangladesh, this approach will have broader applicability in a variety of socioecological systems in developing countries.

## Farmer mental models and FCM

Mental models are widely used for understanding complex socioecological systems and portraying the knowledge and experience of stakeholders in graphical form. Based on Rouse and Morris (1986), mental models of agroecological systems are defined as the representation of systems, including purpose and form, explanation of system functioning and observed system states, and prediction of future system states. In complex socioecological circumstances of multi-stakeholder contexts, such as the coastal farming systems of Bangladesh, the existence of a broad diversity of perspectives and associated mental models is anticipated. However, these representations would be similar for farmers in similar situations and with corresponding resource availability and production orientation. In agroecologies with diverse resource endowments, the mental models of farmers may be influenced by the structural and functional characteristics of the farming systems in which they operate. For instance, the diversity of farming systems can be represented by typologies that segregate farm households into different farm types, such as structural (e.g., landholding, crops, livestock size) and/or functional variables (e.g., cropping intensity, technology adoption). When farms are grouped into types based on functional and structural features, similar mental models are expected to emerge as a function of farm type.

**Fig. 1:**
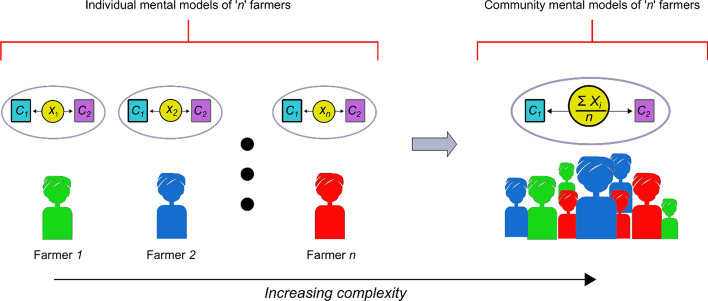
Stylized pictorial overview of grouping individual mental models to form collective mental models used in this study. *C*_*1*_ and *C*_*2*_ are two example concepts represented by cyan and purple boxes, respectively. The relationship between *C*_*1*_ and *C*_*2*_ is indicated by lines with double-sided arrows. The relationship strengths between *C*_*1*_ and *C*_*2*_ for ‘*n*’ number of individual farmers are given by the values *X*_*1*_*, X*_*2*_*,…, X*_*n*_, and the collective community mental model is the average of individual values *X*_*1*_*, X*_*2*_*,…, X*_*n*_*,* given as *(Σ X*_*i*_*)/n*

To arrive at a representation of the mental model of farmers belonging to a particular farm type, a single mental model that represents the group’s understanding in its entirety can be developed through participatory and interactive processes. Interactions between the participants during such processes may, however, not be fully representative. For example, there is extensive literature on the risks of bias in focus groups, where particular individuals may, for example, dominate and skew conversations, thereby reducing the effectiveness of focus groups or similar participatory settings in arriving at an average representation of a particular system (Nyumba et al., 2018). As a result, dominant participants can overly influence and change how less assertive participants express themselves (Jones et al., 2011). Conversely, Gray et al., (2014) demonstrated the validity of an alternative approach based on aggregating and averaging causal relationship strengths perceived by individuals in a group, which can be used to depict a group’s mental model while reducing the risk of bias (Fig. 1). Several subsequent studies modeling the perceptions of socioecological systems (Bunce et al., 2010; Halbrendt et al., 2014; Whitley et al., 2018), in particular coastal systems (Levine et al., 2015), have used aggregation techniques to average individual responses to questions on mental model components and incorporate them into a representative group model.

FCM creates a directional graph that incorporates feedback loops (Kosko, 1986). Like traditional causal concept maps, FCM consists of nodes that represent key ‘concepts’ of the system that are graphically represented by boxes. The links or edges of concepts are given a numerical value, signed as either positive or negative, to represent the nature of their causal relationship. Feedback equips FCM with the ability to assess causal relationships between processes.

We used FCM to model aggregated perspectives on the components and interactions within coastal agroecosystems differentiated by key farm types identified both within and outside the polders in central coastal Bangladesh. This approach should elucidate farmers’ perceived relationships between agroecosystem component dynamics and production risks, irrigation and water management systems, market structures, household priorities, and external development interventions.

## Methodology

### Study area

**Fig. 2:**
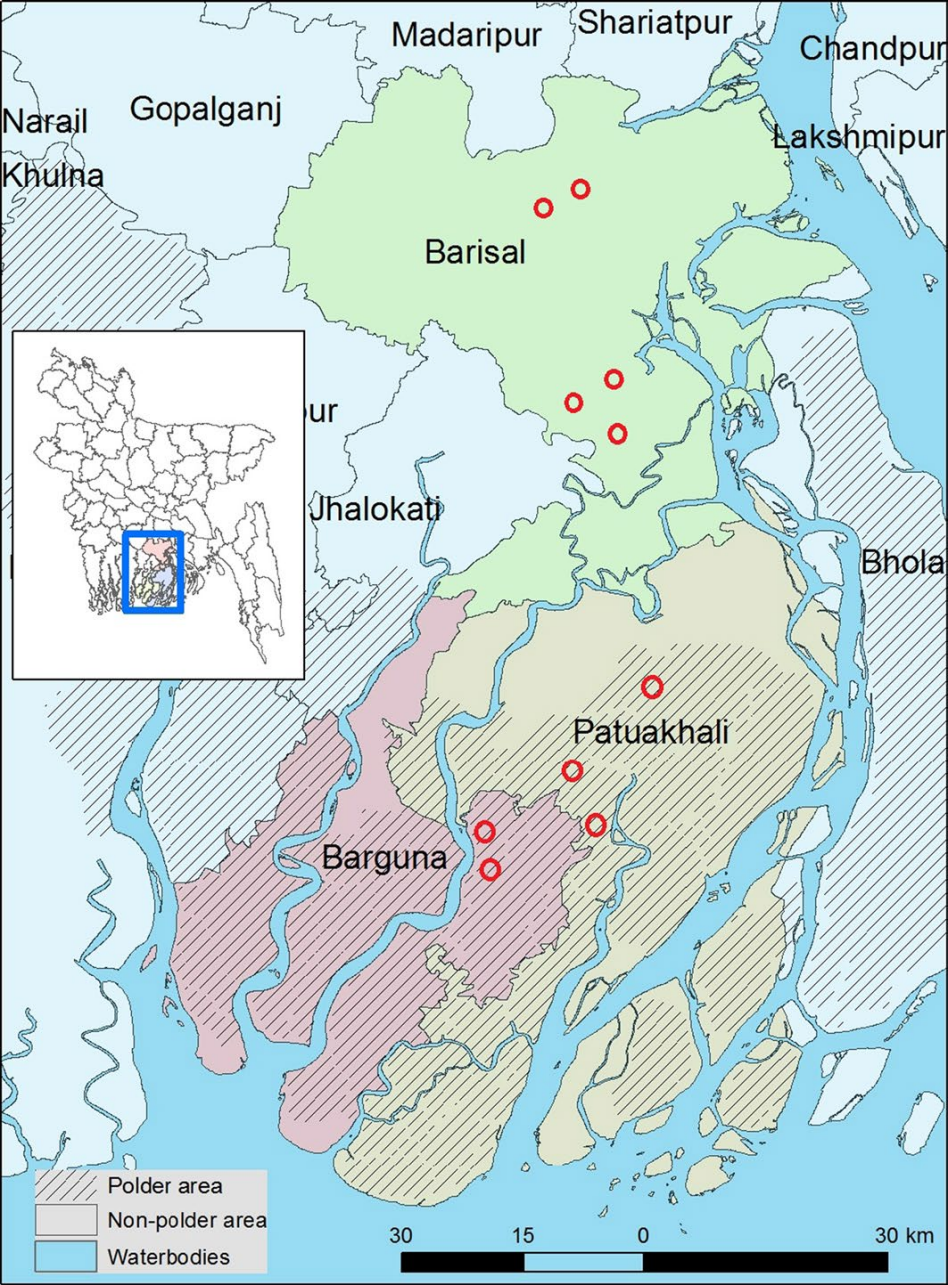
Map of the study districts showing the location of surveyed farmer communities, denoted by red circles

The study area comprised the Barisal, Patuakhali, and Barguna districts in south-central Bangladesh (Fig. 2). The region is characterized by a dense network of interconnected rivers and natural canals that flow into the Bay of Bengal. Annual rainfall ranges from 1955 to 2100 mm (BBS, 2013), with a humid subtropical climate. Most soils are medium- to high-textured silty clay loams (SRDI, 2010). The southern-most part of the central coast (Patuakhali, and Barguna districts) is protected by polder embankments constructed from 1960 onwards. Across the Barisal, Patuakhali, and Barguna districts, approximately 70% of the households in the polders and 59% of the households outside the polders are engaged in farming (BBS, 2013). Most households are engaged in rainfed cropping in the *kharif* (mid-March to mid-November) and drier winter *rabi* (mid-November to mid-March) seasons. *Kharif* sowing coincides with the onset of monsoon and is further divided into pre-monsoon *kharif-1* (mid-March to mid-July) during which local ‘*aus*’ rice varieties are grown, and monsoon *kharif-2* (mid-July to mid-November) when *aman* rice is grown. The *rabi* season falls in the dry winter period, when farmers within polders grow pulses primarily, while farmers in non-polder areas cultivate pulses, mustard, and, to a lesser extent, vegetables. Irrigated *rabi* season rice production, known as *‘boro,’* occurs in select areas proximal to water sources.

### Data collection

Two districts within polders (Patuakhali and Barguna) and a single district outside polders (Barisal district) were selected for the study (Fig. 2), due to their potential for crop intensification and surface water irrigation in coastal Bangladesh (Aravindakshan et al., 2020; Krupnik et al., 2017). Based on the discussions with the experts from the Bangladesh Agricultural Research Institute (BARI) and a local NGO (Bangladesh Development Society (BDS)), five villages each from within and outside the polder areas were selected through a simple random procedure for the FCM survey. This was followed by a non-probability purposive sampling to select the respondents’ from a list of households available with the NGO: BDS, who works with farm households in the area. Out of the total of 250 samples (25 each from the 10 surveyed villages), ten samples were removed due to incomplete surveys. A final sample of 120 HHs each within and outside polders were selected such that at least 5% of all HHs in each of the selected villages were sampled as advised by Turner (2003). The surveys were administered during October–December, 2016. Farm typology variables, including farm structural and functional characteristics, household resource endowments, agricultural management information, on- and off-farm income data, and biophysical, socioeconomic, and demographic attributes were collected alongside questions on FCM concepts and strength of their relationships between them using Likert scale as explained in Sect. 3.3.2.

### Analytical approaches

The analytical approach for the analysis of farmer cognition of farming systems and causal effects of proposed interventions for food security and cropping systems intensification consisted of three discrete steps outlined below.

#### Characterization of farming systems

We used principal component analysis (PCA) and cluster analysis to characterize the farming systems (see Alvarez et al., 2018) and farm households based on their structural (resource endowment) and functional (production and land use objectives/livelihood strategies) characteristics (Kuivanen et al., 2016). The variables used in the farm typology construction are provided in the Supplementary Material (Table S1). Agglomerative hierarchical clustering incorporating Ward’s minimum-variance method was undertaken on the PCA (PC scores) to identify clusters. Ward’s method minimizes within-cluster variation by comparing two clusters using the sum of squares between two clusters, summed over all variables (Alvarez et al., 2018).

#### Identification of farming system concepts and potential drivers

Farmers’ cognition of farming system concepts and potential interventions/drivers of change were identified by focus group discussions (FGDs) with three groups of farmers belonging to different farm types, as identified from Step 1 (Sect. 3.3.1). Sixty-five randomly selected farmers in Barisal, Patuakhali, and Barguna districts participated in the FGDs. The FGDs were administered (during August–September 2016) using a semi-structured questionnaire to identify and discuss the farmers’ present and previous experiences with double cropping, irrigation, crop diversification, and production risks, and to identify relevant internal and external factors perceived as influencing their farming system. We identified the most common themes in the responses given by farmers in the FGD, which were divided into concepts and given titles to each thematic response. Then, the relationships between the concepts were identified based on the qualitative information given by FGDs.

During the FCM surveys, the farmers were asked to identify whether they perceived relationships between map concepts. If respondents indicated their belief in a relationship, they were asked to quantify the degree to which these relationships affected the concepts using a categorical scale translated as *very, moderately, or slightly influential*. These data were used to determine the weights of causal relationships, according to a 7-point Likert scale, to establish the positive or negative influence and degree of strength for each causal relationship represented in the concept map. Based on this information and data, distinct fuzzy cognitive maps were developed in FuzzyDANCES Software—part of a multi-scale agricultural modeling framework called COMPASS (Groot et al., 2012) (Box S1 in the supplementary material)—by aggregating individual measures assigned by farmers for farm types within and outside the polders. A *Kruskal–Wallis H* test was used to compare the causal relationships (relationship weights) between concepts in the FCM of each farm type within a study environment.

#### Simulation of interventions and winding stairs algorithm

The dynamics of the states of the concepts in an FCM can be assessed quantitatively by iterative matrix multiplication using the program FuzzyDANCES (Box S1 in the supplementary material). A balanced FCM will lead to equilibrium values for the concept state values (Kosko, 1986). We used a multiplication function wherein the new state is independent of the current state of the concept (e.g., Stach et al., 2005).

1 $$A_{i} \left( {t + 1} \right) = \mathop \sum \limits_{{\begin{array}{*{20}c} {j \ne j} \\ {j = 1} \\ \end{array} }}^{N} w_{ji} \times A_{j} \left( t \right)$$

where *t* is the iteration number, *A*_*i*_*(t),* and *A*_*i*_*(t* + *1)* are the state values of concept *i* at iterations *t* and *t* + *1*, and *w*_*ji*_ is the weight of the relationship between concepts *j* and *i*.

The winding stairs (WS) algorithm is based on the Monte Carlo sensitivity analysis but performs a factorial analysis of the effect of multiple parameters on the performance of response variables within modeled systems (Chan et al., 2000; Jansen et al., 1994), in our case represented by the FCM. This allows us to analyze the sensitivity of target performance indicators in the system (i.e., selected concept state variables) to changes in the weight of external drivers or relationships in the system. Thus, the parameters of the sensitivity analysis can be either two or more state values of system drivers, or the values of a subset of the causal relationships between concepts within the FCM. A scalar model output *Y*_*i*_, representing one of the target state values in the FCM, depends on the influences of inputs factor vectors *X*_*1*_, *X*_*2*_,…, *X*_*k*_, following Eq. 2, that we treat as random variables because they vary about nominal values that are unknown (Chan et al., 2000).

2 $$Y_{i} = f\left( {X_{1} ,X_{2} , \ldots ,X_{k} } \right)$$

The function *f* is deterministic and, in this case, the result of the matrix multiplications of the FCM to the equilibrium state. The systematic sampling applied allows the variability of *f* to be expressed as its variance, and the proportions of the variance caused by the input factors *X*_*1*_, *X*_*2*_, … *X*_*k*_. These factors are randomly sampled in cyclical order, with new values {*x*_*11*_, *x*_*21*_, … *x*_*k1*_}, for the first step of cycle 1. In this step, *x*_*11*_ is randomly adjusted, in the second *x*_*21*_, etc. Thus, each cycle contains *k* steps that constitute one WS sample or ‘winding’ (Jansen et al., 1994). The number of WS samples generated (*l*) can be set as a parameter of the algorithm. The total number of observations generated is *N* = *k* × *l*.

We used the WS analysis to decompose model variance into the first-order sensitivity index (SVI) and total sensitivity index (TSI) (Chan et al., 2000). The SVI is defined as the variance reduction due to fixing factor *X* while varying the other factors (also denoted as top marginal variance) (Jansen et al., 1994). The TSI is, conversely, the variance caused when only *X*_*i*_ is uncertain (bottom marginal variance; Jansen et al., 1994). The TSI measures the contribution of an input factor *X*_*i*_ to the total model output variation (Chan et al., 2000; Homma & Saltelli, 1996). We analyzed two outputs of the WS sensitivity analysis, including (1) changes in the state values of the selected performance indicators of intensification listed in Sect. 2.5 in response to modeled perturbations in the external drivers and (2) the TSI of the indicators to the external drivers. The sensitivity analysis of drivers in this study involved a resampling procedure where the driver state values are manipulated from a start value of [0.5], to a maximum of 1.0 and a minimum of 0, after setting it as an objective, followed by running the WS algorithm through 1000 windings. The winding stairs method involves computing the model outputs after each drawing of a new value for an individual parameter (driver) and building a WS-matrix.

## Results

### Characterization of farming systems and farm typology

**Fig. 3:**
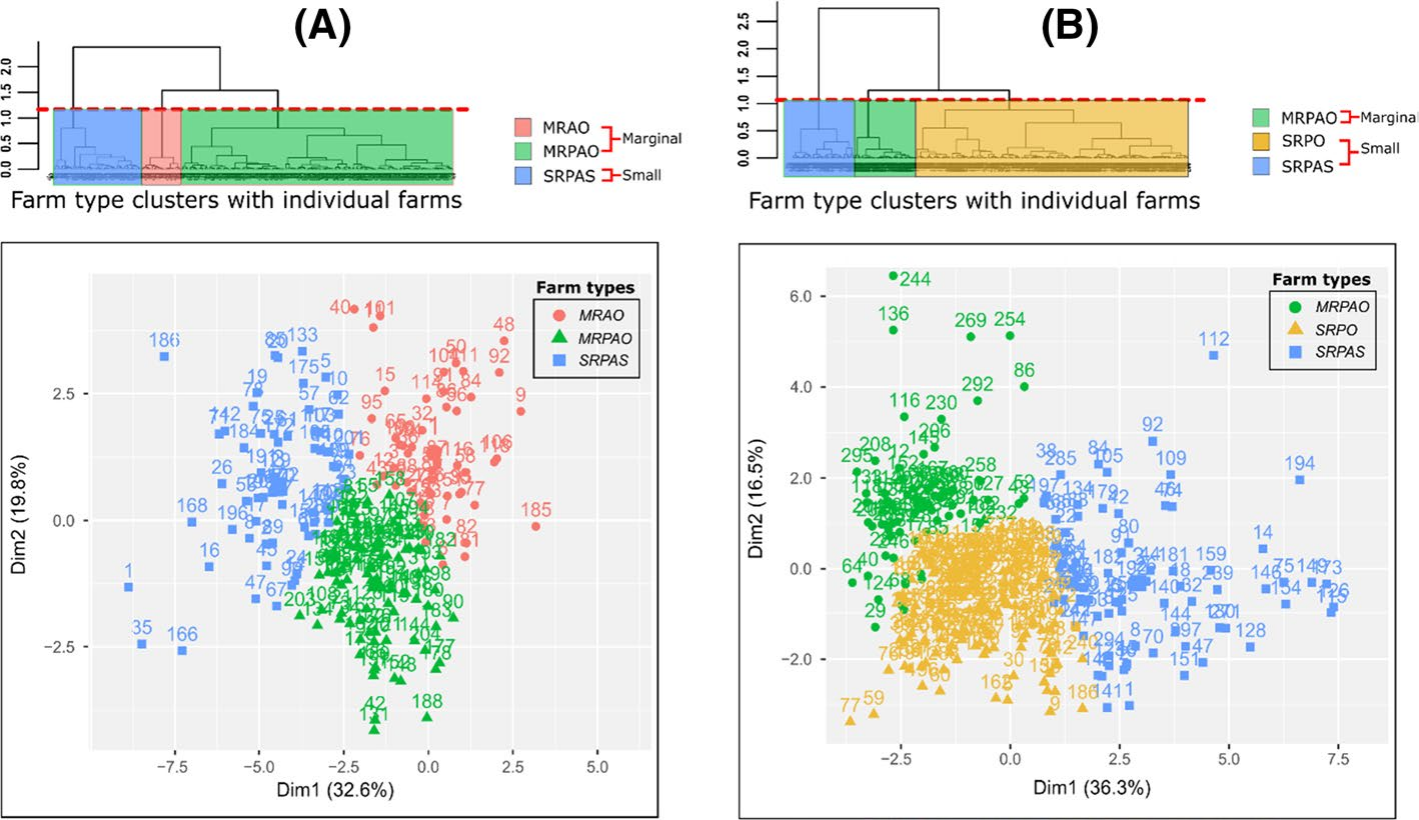
Results of the typology analysis for farms (**a**) outside and (**b**) within polders along the first two principal components. MRAO = Marginal farms with rice–aquaculture systems and off-farm income; MRPAO = Marginal farms with rice–pulse–aquaculture systems and off-farm income; SRPAS = Small sharecropping farms with rice–pulse–aquaculture systems; SRPO = Small farms with rice–aquaculture systems and off-farm income

The overview of typology variables and their descriptive statistics is provided in the supplementary material (Table S1). Analysis of the farm typology data yielded a multivariate classification of distinct farm typologies segregated by those located outside (OP) or within (WP) polders, each resulting in three distinct farm types (Fig. 3a and b; Table 1). The detailed description of the identified farm types is given in the supplementary material (Box S2). The dendrograms and cut-off points based on dissimilarity for farms located within or outside polders are also shown in Fig. 3. PCs with eigenvalues ≥ 1 explained more than 70% of the variability in farm typology data, both within and outside polders. The first four PCs explained cumulative variability of 72% and 73% for farmers within and outside polder areas, respectively.

**Table 1:** Farm types identified in the study area

Outside polders		Within polders	
Cluster OP-1	Marginal farms with rice–aquaculture systems and off-farm income (MRAO)	Cluster WP-1	Marginal farms with rice–pulse–aquaculture systems and off-farm income (MRPAO)
Cluster OP-2	Marginal farms with rice–pulse–aquaculture systems and off-farm income (MRPAO)	Cluster WP-2	Small farms with rice–aquaculture systems and off-farm income (SRPO)
Cluster OP-3	Small sharecropping farms with rice–pulse–aquaculture systems (SRPAS)	Cluster WP-3	Small sharecropping farms with rice–pulse–aquaculture systems (SRPAS)

### General structure of FCM

**Fig. 4:**
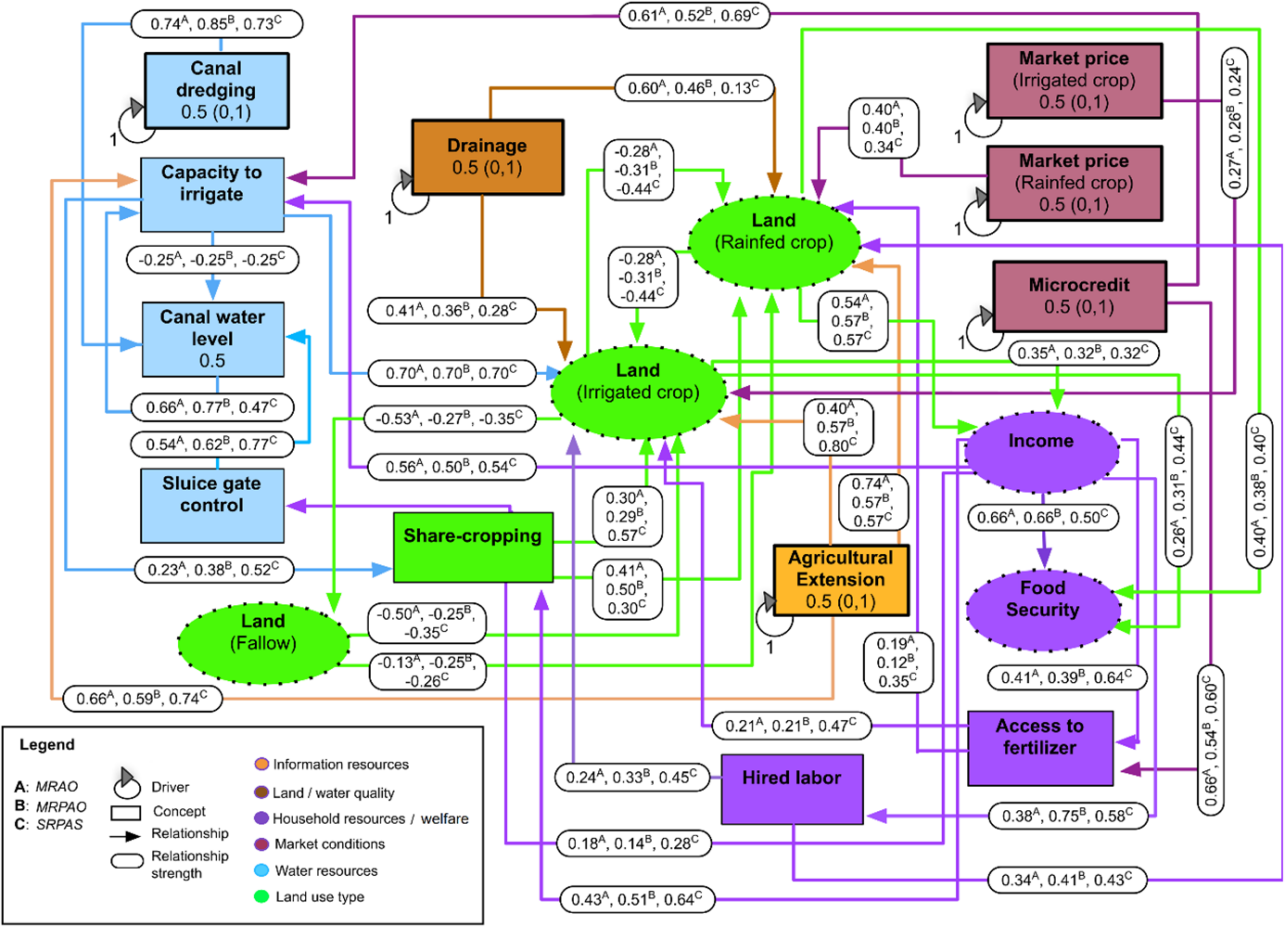
Aggregate irrigation–farm–household system Fuzzy cognitive mapping for outside polder farm types (A) Marginal farms with rice–aquaculture systems and off-farm income, (B) MRPAO= Marginal farms with rice–pulse–aquaculture systems and off-farm income, (C) Small sharecropping farms with rice–pulse–aquaculture systems. The system drivers—identified as external transmitter variables that are subject to state changes (natural or intentional intervention)—are indicated by looped arrows and are outlined in black. To better understand the Fuzzy cognitive mapping, concepts are grouped into color-coded categories based on their function within the system, i.e., information resource, land/water quality, household resources and welfare, market conditions, water resources, and land use types. Values in boxed concepts represent the baseline values applied in the winding stairs sensitivity analysis (WSSA). Values within the box outside boxes correspond to current state while (0, 1) indicates the minimum and maximum range of simulation in the WSSA

**Fig. 5:**
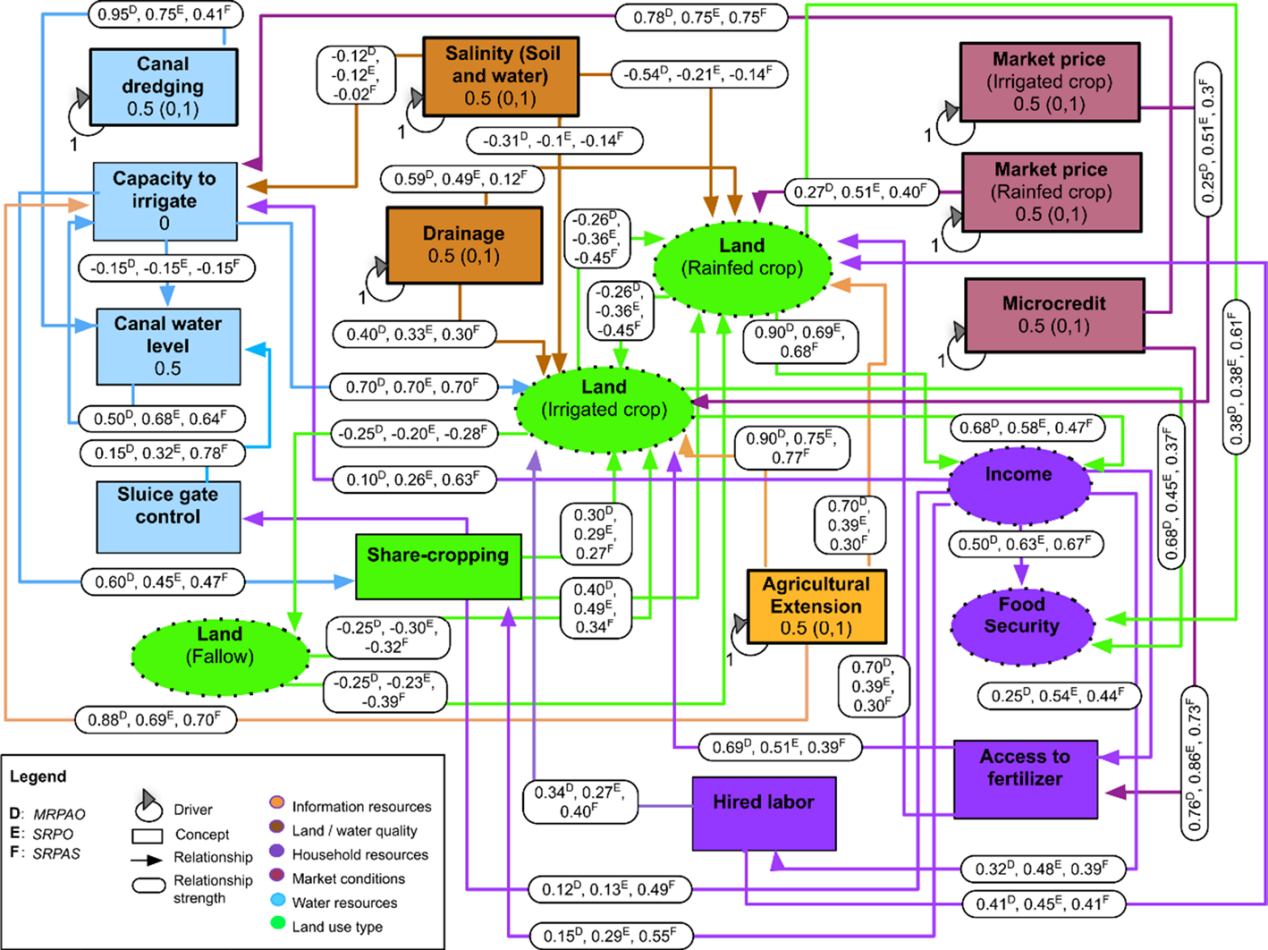
Aggregate irrigation–farm–household system Fuzzy cognitive mapping for within polder farm types: (D) marginal farms with rice–pulse–aquaculture systems and off-farm income, (E) small farms with rice–aquaculture systems and off-farm income, (F) small sharecropping farms with rice–pulse–aquaculture systems. The system drivers—identified as external transmitter variables that are subject to state changes (natural or intentional intervention)—are indicated by looped arrows and are outlined in black. To better understand the Fuzzy cognitive mapping, concepts are grouped into color-coded categories based on their function within the system, i.e., information resource, land/water quality, household resources and welfare, market conditions, water resources, and land use types. Values in boxed concepts represent the baseline values applied in the winding stairs sensitivity analysis (WSSA). Values within the box outside boxes correspond to current state, while (0, 1) indicates the minimum and maximum range of simulation in the WSSA

Six FCMs, each representing the mental models (Fuzzy Cognitive Maps) of farmers belonging to each of the identified farm types, were developed in FuzzyDANCES separately (Box S1 and Figures S2–S7 in the Supplementary Materials). For brevity, we represented FCMs belonging to a particular environment together in a single figure. For example, the three FCMs corresponding to the farm types outside the polders were shown in a single fuzzy cognitive map (Fig. 4) by representing the relationship strength between the concepts of farm types MRAO, MRPAO, and SRPAS using English letters A, B, and C (superscripted above the values assigned to relationship strengths), respectively and similarly, for farm types MRPAO, SRPO, and SRPAS within the polders, using D, E, and F, respectively (Fig. 5). The FCM for each farm type, both within and outside polders, identifies pathways where dry season fallows in the *rabi* (winter) season can be used for cropping to improve household income and food security. The concepts and drivers, as conceptualized by farmers in coastal areas of south-central Bangladesh during the FGDs, are provided in Table 2. Concepts that frequently emerged in the FGDs and were considered very important by the community and the authors were included for the construction of each FCM. Selected concepts had robust causal relationships with household income, food security, and crop management. The baseline structure of the FCM for each farm type outside (Fig. 4) and within (Fig. 5) polders shows the direction, strength, and sign for each of the causal relationships identified and averaged across the study. A brief overview of these grouped concepts is provided below.

**Table 2:** Description of the concepts and drivers in the baseline fuzzy cognitive mapping, as conceptualized by farmers in coastal areas of south-central Bangladesh, during focus group discussions

Concept	Concept type	Concept group/indicators	Description
Extension	Driver	Information source	Agricultural advisory or information services received by farmers from public or private agencies
Drainage	Driver	Land/water quality	Removal of excess water present in farm fields via hand- or back-hoe dug drainage channels. Drainage is necessary in low-lying areas of coastal Bangladesh, where waterlogging can hamper production of mungbean, lathyrus (grass pea) and vegetables
Salinity	Driver	Land/water quality	Increasing water salinity that intrudes into cropland and affects soils
Income	Receiver/transmitter	Sustainable intensification (household welfare)	Total household income of a farm household, comprising farm and non-farm income, and remittances
Food security	Receiver	Sustainable intensification (household welfare)	Condition in which all members of a household, at all times, have physical and economic access to sufficient food to meet their dietary needs and food preferences for an active and healthy life
Fertilizer access	Receiver/transmitter	Household resources	Farmers’ access to outlets selling fertilizers and the financial means to do so
Hired labor	Receiver/transmitter	Household resources	Hired laborers paid by the farmer to conduct farm operations (e.g., land preparation, transplanting, weeding, harvesting)
Market prices of irrigated crops	Driver	Market conditions	Farm-gate price paid for irrigated crops (i.e., *boro* rice, maize, wheat)
Market prices of rainfed crops	Driver	Market conditions	Farm-gate price paid for rainfed crops (i.e., mungbean and lathyrus (grass pea))
Micro-credit	Driver	Market conditions	Access to and ability of the farmer to avail financial loans for funding crop production activities
Canal dredging	Driver	Water resources	Excavation and removal of silt and sediments accumulated in irrigation canals to improve the flow of irrigation water to farmers’ fields. Dredging is usually carried out by government or non-governmental agencies
Capacity to irrigate	Receiver/transmitter	Water resources	Ability of the farmer to irrigate crop fields, further determined by access to finance and irrigation service provision
Canal water level	Receiver/transmitter	Water resources	Level of water flowing through irrigation canal during the dry *rabi* season. Farmers consider a threshold water level of 0.6 m as necessary for pumping with low-lift pumps. It is not feasible to pump water when the level drops below this threshold
Sluice control	Receiver	Water resources	Management of sluice gates to open or close canals to allow water flow. Often controlled by individuals or groups in a community
Irrigated crops	Receiver/transmitter	Land use type	*Boro* rice, wheat, and maize are considered *rabi* season crops that require irrigation. *Boro* rice is cultivated in some parts of the Barisal division; wheat and maize are relatively new to the area
Rainfed crops	Receiver/transmitter	Land use type	Mungbean and lathyrus (grass pea); commonly cultivated without irrigation in the *rabi* season, after harvest of ‘*aman*’ rice
Fallow land	Receiver/transmitter	Land use type	Land left uncultivated during the dry *rabi* season, which could potentially be utilized for a second, irrigated crop
Share cropping	Receiver/transmitter	Land use type	Practice in which a landowner permits a farmer to use the land for crop production, in return for a share of the crops harvested. Commonly, ~ 30–40% of the harvest is shared by the tenant farmer with the landowner. Most farmers in the Barisal division are share-croppers

*Note*: Concept groups/categories are based on Smith et al. (2017) and discussion with key informants from BARI

#### Information resources

‘Information resources’ refer to sources of information and the provision of advice that assists farmers in cropping and marketing decisions. Although this may be open to a wide variety of interpretations, the farmers in the FGDs clarified that they conceptualize this concept as agricultural extension. In our study area, field officers from the Department of Agricultural Extension are primary sources of information on crop and input management strategies.

#### Land/water quality

Drainage and salinity are the concepts included in the ‘land and water quality’ category. ‘Salinity’ is conceptualized by farmers in terms of both soil and water salinity that affects crop production. ‘Drainage’ is conceptualized as the removal of excess water present in the fields through drainage channels. While rice can withstand waterlogging, but not extended submergence, drainage is necessary for low-lying areas of coastal Bangladesh, where waterlogging can hamper the production of pulses, and vegetables. Outside the polders, salinity problems were not present. So only drainage was included in the land and water quality category for farms outside polders, while for those inside polders both salinity and drainage were included.

#### Household resources and welfare

Household resources consist mainly of the income from farm and non-farm activities and remittances, while household welfare includes its command over market and non-market goods and services. We included the concepts ‘household income,’ ‘food security,’ and ‘households’ ability to purchase or access fertilizers and hired labor’ in the ‘household resources and welfare.’ Income and food security of the households are directly related to the intensification of crops in southern Bangladesh, whether it is irrigated or rainfed. A change in income can have a direct effect on a household’s ability to access inputs and purchase irrigation services.

#### Market conditions

The concept of ‘market conditions’ is based on farmers’ market access and the price of the final product. In the absence of sufficient storage facilities, demand and access to markets can exert a strong influence on cropping patterns by increasing double cropping (Aravindakhshan et al., 2020). In the FGDs, farmers clarified that irrigated crop prices are conceptualized as the market price of *boro* rice, while the rainfed crop price is conceptualized as the market price of rainfed pulses, such as mungbean.

#### Water resources

‘Water resources’ include numerous concepts in the literature; here, water resources in FCM involved four concepts that were considered important in the FGDs—'canal dredging,’ ‘capacity to irrigate,’ ‘canal water level,’ and ‘sluice gate control.’ Farmers clarified that the concept of canal dredging refers to the excavation and removal of silt and sediments accumulated in canals from which surface water can be withdrawn for irrigation. Siltation can prevent the regular flow of water adjacent to farmers’ fields, particularly in the dry season. Canal dredging requires specialized equipment at substantial cost and cannot typically be borne by farmers alone. It is usually carried out by governmental agencies, such as the Bangladesh Agricultural Development Corporation or the Local Government Engineering Department.

The ‘capacity to irrigate’ is the ability of the farmer to irrigate their crop fields on a timely and efficient basis, which has an influence on crop productivity. In the FGDs, farmers indicated that their ability to irrigate is, in turn, affected by the availability of micro-credit to purchase fuel for pumps or contract a pump owner to provide irrigation to their fields. However, the farmers clarified that even if they were willing to purchase irrigation services, they do not consider it feasible to pump water when the water level in canals drops below a threshold of 0.6 m depth. As such, canal water levels were clarified during surveys as the average level of water flowing through and available in canals during peak times for crop irrigation demand in the dry *rabi* season. Finally, the water level and flow in canals are typically regulated by the opening and closing of a movable sluice gate (Krupnik et al., 2017). The concept of sluice gate control refers to an adjustable gate allowing water to flow through it.

Land that is fallowed in the dry season can, in principle, be cropped during the dry *rabi* season, when water resources (e.g., surface water) are available and farmers are willing to invest in irrigation. When and where irrigation sources are not well developed, or in situations where farmers are unwilling to invest in irrigation, rainfed crops or crops established with residual soil moisture after the monsoon can be an alternative. These crops—typically pulses—can be used to convert fallows and increase cropping intensity. Though small quantities of irrigation water can considerably improve the yield of these crops, the cost of irrigation pumping service provision or de-silting canals may prevent wide-scale use among farmers.

#### Land use types

In the current study, the concept of ‘land-use types’ consists of irrigated cropland, rainfed cropland, and fallow. While irrigated crops mainly involve *boro* rice, wheat, and maize with irrigation from canals or rivers, in the FGDs, the farmers grouped rainfed crops into a category comprising unirrigated pulses, such as mungbean, lentil, and grass pea, which are typically established using only residual soil moisture. Farmers also clarified that the concept of ‘fallow land’ is farmland that remains uncropped during the *rabi* (winter) season. Reasons for land fallowing may include farmers’ inability to invest in irrigation and fertilizer inputs, lack of irrigation infrastructure, canal water level below the threshold for irrigation, early withdrawal of monsoon rains leading to insufficient soil moisture for sowing winter crops, waterlogging and excessive moisture, and lack of suitable crop varieties for late planting in the *rabi* season. In addition to the various land-use types, ‘sharecropping’ is another important concept identified in FGDs, which includes arrangements for a landowner to permit a farmer (who becomes a tenant in terms of sharing the land for cropping) to use the land in return for a share of the crops produced on the landowner’s portion of land. Survey data indicated that roughly 30–40% of the crop harvest is shared by the tenant farmer with the landowner.

### Similarities and dissimilarities between FCM within and outside polders

Network structures for the combined FCM within a study environment (i.e., within polders or outside polders) were relatively uniform when not distinguished by farm typology (Table 3). The sample farm types tended to agree with respect to FCM concepts, except for the driver ‘soil and water salinity’ for the combined FCM within the polder area, which was not identified by farmers outside polders. In total, 17 concepts and 35 relationships were included in each farm-type FCM outside polders. The farm types within polders included 18 concepts and 38 relationships. The concepts were similar, and the strength of relationships between concepts only varied slightly among farm types within a study environment.

**Table 3:** Network metrics for baseline concept maps of farming systems in south-central Bangladesh, disaggregated by study environments and farm types

Metrics	Outside polder area			Within polder area		
	MRAO	MRPAO	SRPAS	MRPAO	SRPO	SRPAS
Number of concepts	17.00	17.00	17.00	18.00	18.00	18.00
Number of relations	44.00	44.00	44.00	47.00	47.00	47.00
Density (clustering coefficient)	0.16	0.16	0.16	0.15	0.15	0.15
Hierarchy index	0.07	0.07	0.08	0.07	0.07	0.06
Number of transmitters	0.00	0.00	0.00	0.00	0.00	0.00
Number of receivers	1.00	1.00	1.00	1.00	1.00	1.00
Number of ordinary concepts	16.00	16.00	16.00	17.00	17.00	17.00

*Notes: MRAO* marginal farms with rice–aquaculture systems and off-farm income, *MRPAO* marginal farms with rice–pulse–aquaculture systems and off-farm income, *SRPAS* small sharecropping farms with rice–pulse–aquaculture systems, *SRPO* small farms with rice–aquaculture systems and off-farm income

*Kruskal–Wallis H* tests were used to compare the relationships *strengths* between concepts in the combined FCM from within or outside polder areas. First, the combined FCM of farmers within polders was analyzed without differentiating the sample into farm types. When comparing the combined FCM of the two locations (i.e., within and outside polders), of the 35 relationships in each FCM, 12 showed significant differences (Table 4). For example, the relationships between the price of irrigated crops and irrigated crop area (*P* = 0.001), micro-credit and the capacity to irrigate (*P* = 0.000), micro-credit and fertilizer access (*P* = 0.000), agricultural extension and irrigated crop area (*P* = 0.002), canal dredging and canal water level (*P* = 0.000), irrigated crop area and household income (*P* = 0.027), irrigated crop area and food security (*P* = 0.000), area of irrigated crops and fallow land (*P* = 0.000).

**Table 4:** Difference in fuzzy cognitive mapping of farming communities within and outside polder areas of coastal Bangladesh

Relationships between concepts in the system	Outside polders		Within polders		*P-*value
	Mean	SD	Mean	SD	
Market prices (irrigated crops)—irrigated crops area	0.257	0.229	0.410	0.338	0.001**
Market prices (rainfed crops)—rainfed crops area	0.385	0.267	0.448	0.315	0.385
Micro-credit—capacity to irrigate	0.588	0.355	0.756	0.341	0.000***
Micro-credit—fertilizer access	0.576	0.366	0.807	0.310	0.000***
Agricultural extension—capacity to irrigate	0.651	0.346	0.712	0.353	0.101
Agricultural extension—irrigated crops area	0.616	0.371	0.769	0.251	0.002**
Agricultural extension—rainfed crops area	0.597	0.357	0.554	0.396	0.547
Drainage—irrigated crops area	0.343	0.221	0.326	0.214	0.555
Drainage—rainfed crops area	0.381	0.362	0.368	0.348	0.806
Canal dredging—canal water level	0.794	0.373	0.639	0.379	0.000***
Irrigated crops area—rainfed crops area	–0.344	0.551	–0.387	0.524	0.631
Irrigated crops area—household income	0.317	0.226	0.412	0.289	0.027*
Irrigated crops area—food security	0.291	0.226	0.460	0.305	0.000***
Irrigated crops area—fallow	–0.467	0.310	–0.345	0.282	0.000***
Rainfed crops area—irrigated crops area	–0.344	0.551	–0.387	0.524	0.631
Rainfed crops area—household income	0.560	0.169	0.685	0.193	0.000***
Rainfed crops area—food security	0.432	0.328	0.582	0.279	0.002**
Rainfed crops area—fallow	–0.463	0.319	–0.431	0.340	0.304
Fallow—irrigated crops area	–0.454	0.308	–0.287	0.278	0.000***
Fallow—rainfed crops area	–0.388	0.301	–0.343	0.292	0.183
Access to fertilizer—irrigated crops area	0.292	0.286	0.474	0.321	0.000***
Access to fertilizer—rainfed crops area	0.203	0.266	0.348	0.245	0.000***
Hired labor—irrigated crops area	0.315	0.313	0.373	0.322	0.183
Hired labor—rainfed crops area	0.371	0.281	0.437	0.254	0.241
Sluice gate control—canal water level	0.653	0.426	0.688	0.443	0.449
Canal water level—capacity to irrigate	0.689	0.371	0.668	0.378	0.794
Capacity to irrigate—sharecropping	0.398	0.411	0.475	0.416	0.174
Sharecropping—irrigated crops area	0.314	0.165	0.285	0.163	0.228
Sharecropping—rainfed crops area	0.474	0.272	0.427	0.293	0.170
Household income—capacity to irrigate	0.521	0.325	0.465	0.344	0.264
Household income—access to fertilizer	0.468	0.391	0.512	0.417	0.377
Household income—hired labor	0.464	0.357	0.466	0.373	0.914
Household income—sluice gate control	0.191	0.358	0.352	0.378	0.002**
Household income—sharecropping	0.539	0.408	0.479	0.405	0.264
Household income—food security	0.689	0.327	0.643	0.350	0.346

*Mean* mean values of relationship weights, *SD* standard deviation

*Notes*: Differences between fuzzy cognitive maps assessed by *Kruskal–Wallis* H test; *, **, and *** indicate significance at the 10%, 5%, and 1% levels, respectively

Of the 35 relationships in each of the farm type specific FCM constructed representing the perceptual models of farmers outside the polders, eight significantly differed in their relationship strengths (see Supplementary Material, Table S2), including agricultural extension and irrigated crop area (*P* = 0.004), drainage and rainfed crop area (*P* = 0.000), fallow and rainfed crop area (*P* = 0.002), access and fertilizer to rainfed crop area (*P* = 0.002), and hired labor and irrigated crop area (*P* = 0.041). In addition, significant differences were found in farmers’ mental models, outside the polders, including sluice control and the capacity to irrigate (*P* = 0.050), canal water level and capacity to irrigate (*P* = 0.053), and household income and ability to access (purchase) to fertilizer (*P* = 0.036).

Within polders, FCM significantly differed with farm typology for 15 of the 38 perceived relationships (Supplementary Material, Table S3), being market prices of irrigated crops and irrigated crop area (*P* = 0.000), market prices of rainfed crops and rainfed crop area (*P* = 0.009), drainage and rainfed cropped area (*P* = 0.000), canal dredging and canal water levels (*P* = 0.000), irrigated crop area and food security (*P* = 0.016), irrigated crop area and land fallowing (*P* = 0.002), rainfed crop area and food security (*P* = 0.039), rainfed crop area and fallow land area (*P* = 0.004), fallowed land and rainfed crop area (*P* = 0.005), fertilizer access and irrigated crop area (*P* = 0.023), hired labor and irrigated crop area (*P* = 0.031), sharecropping and rainfed crop area (*P* = 0.001), household income and farmers’ capacity to irrigate (*P* = 0.000), household income and hire labor (*P* = 0.006), and household income and ability to control sluice gates (*P* = 0.001).

### Dynamics of FCM

**Fig. 6:**
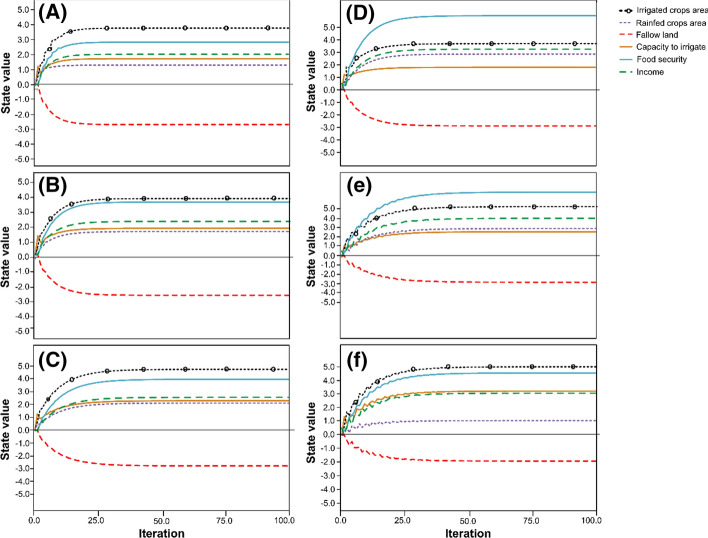
Stabilization of the state values of each concept in the fuzzy cognitive mapping of farm types outside polders: (**a**) MRAO = marginal farms with rice–aquaculture systems and off-farm income, (**b**) MRPAO = marginal farms with rice–pulse–aquaculture systems and off-farm income, (**c**) SRPAS = small sharecropping farms with rice–pulse–aquaculture systems, and within polders, (**d**) MRPAO = marginal farms with rice–pulse–aquaculture systems and off-farm income, (**e**) SRPO = small farms with rice–aquaculture systems and off-farm income and (**f**) SRPAS = small sharecropping farms with rice–pulse–aquaculture systems after 100 iterations

The FCMs stabilized between 20 and 50 iterations (Fig. 6), although several additional iterations up to 100 would ideally be performed to ensure the FCM equilibrium (Kok, 2009).

For farm types outside polders, the highest steady-state values for SI indicator concepts above and below the origin (0.0) are shown by irrigated crop area (3.76, 3.91, and 4.75 for MRAO, MRPAO, and SRPAS, respectively) and fallow land (− 2.69, − 2.80, and − 2.76 for MRAO, MRPAO, and SRPAS, respectively). For MRPAO and SRPO within polders, the highest steady-state values were obtained for food security (5.93 and 6.81, respectively), with the maximum steady-state value for SRPAS attributed to irrigated crop area (5.01). Similar to the farm types within polders, the lowest steady-state values for types outside the polders were obtained for fallow land (i.e., − 2.89, − 2.84, and − 1.91 for MRPAO, SRPO, and SRPAS, respectively).

### Sensitivity of SI indicators to interventions

The potential effect of the different policy interventions represented in the FCM—using the concepts of agricultural extension, micro-credit access, drainage, market prices of irrigated and rainfed crops, and canal dredging as indicator concepts, were explored using sensitivity analysis with the WS algorithm (Tables 5 and 6). Both within and outside polder areas, the highest TSI was observed for effects of extension on changes in other concepts in the map, particularly food security and income (SI indicators). Outside polders, the concepts of drainage and micro-credit were also influential (Table 5); within polders, the availability of micro-credit appears to affect farmer perceptions of SI indicators more than drainage (Table 6).

**Table 5:** Total sensitivity index (%) of interventions/drivers on system indicators, such as food security, farmer income, fallow land, farm area under irrigated (IRC) and rainfed crops (RFC), and capacity to irrigate in fuzzy cognitive mapping of farm types outside the polders

Drivers	Capacity to irrigate			Fallow land			Irrigated crops			Rainfed crops			Food security			Income		
	MRAO	MRPAO	SRPAS	MRAO	MRPAO	SRPAS	MRAO	MRPAO	SRPAS	MRAO	MRPAO	SRPAS	MRAO	MRPAO	SRPAS	MRAO	MRPAO	SRPAS
Agricultural extension	**52.41**	**48.10**	**55.51**	**46.40**	**47.40**	**58.20**	**47.00**	**50.70**	**60.00**	**38.40**	**35.80**	**50.90**	**45.80**	**45.70**	**57.30**	**45.80**	**45.10**	**57.41**
Micro-credit	**20.32**	**15.6**	**25.2**	**8.10**	**6.70**	**16.80**	**12.20**	**9.20**	**19.20**	1.30	2.40	11.91	7.10	**5.90**	**16.00**	**6.90**	5.50	**16.11**
Drainage	**14.81**	**19.4**	**5.62**	**39.80**	**33.50**	**13.30**	**33.80**	**26.60**	**10.80**	**49.70**	**46.20**	**17.40**	**41.60**	**35.90**	**14.10**	**41.60**	**36.90**	**13.90**
Current market prices of rainfed crops	1.60	2.41	2.43	3.60	4.44	4.70	1.40	1.70	1.50	**12.50**	**13.50**	**14.80**	4.50	5.70	6.10	4.60	**6.30**	5.91
Current market prices of irrigated crops	0.21	0.2	0.11	0.90	0.91	0.30	2.10	2.1	0.80	0.10	0.00	0.00	0.60	0.70	0.20	0.60	0.50	0.20
Canal dredging	9.00	14.10	5.00	1.70	4.90	1.60	3.30	7.50	2.31	0.00	1.00	0.50	1.32	4.10	1.30	1.31	3.70	1.42

*Notes*: Jansen (winding stairs) method performed using the winding stairs sensitivity algorithm (WSSA). Total sensitivity index (TSI) gives total order variance of a driver on the FCM model output—*ceteris paribus*. The three drivers with the maximum TSI percentage for each indicator for each farm type are indicated in bold

*MRAO* marginal farms with rice–aquaculture systems and off-farm income, *MRPAO* marginal farms with rice–pulse–aquaculture systems and off-farm income, *SRPAS* small sharecropping farms with rice–pulse–aquaculture systems, *SRPO* small farms with rice–aquaculture systems and off-farm income

**Table 6:** Total sensitivity index (%) of interventions/drivers on system indicators such as food security, farmer income, area fallow land, farm area under irrigated (IRC) and rainfed crops (RFC), and capacity to irrigate in the fuzzy cognitive mapping of farm types within the polders

Drivers	Capacity to irrigate			Fallow land			Irrigated crops			Rainfed crops			Food security			Income		
	MRPAO	SRPO	SRPAS	MRPAO	SRPO	SRPAS	MRPAO	SRPO	SRPAS	MRPAO	SRPO	SRPAS	MRPAO	SRPO	SRPAS	MRPAO	SRPO	SRPAS
Agricultural extension	**56.20**	**47.60**	**65.50**	**59.00**	**47.70**	**67.80**	**62.50**	**51.50**	**69.90**	**56.10**	**38.60**	**51.60**	**60.10**	**47.00**	**68.10**	**59.80**	**46.00**	**68.20**
Micro-credit	**26.50**	**34.40**	**29.70**	**13.80**	**26.80**	**21.10**	**21.40**	**29.90**	**25.20**	**21.70**	**20.40**	**9.90**	**15.30**	**26.30**	**21.40**	**15.90**	**25.50**	**21.60**
Drainage	3.50	6.20	1.50	**17.31**	**11.80**	2.53	**8.40**	**8.60**	2.90	**10.70**	16.90	1.20	**15.30**	**12.30**	2.51	**14.90**	**13.00**	2.50
Current market prices of rainfed crops	0.50	5.00	1.50	2.90	8.50	**10.20**	0.50	3.80	**3.50**	4.50	**18.70**	**36.00**	2.30	9.20	**9.60**	2.50	10.41	**9.21**
Current market prices of irrigated crops	0.00	0.50	0.30	0.10	1.50	0.40	0.60	3.40	1.40	0.00	0.00	0.40	0.20	1.30	0.50	0.11	1.01	0.50
Canal dredging	**10.80**	**6.40**	**5.40**	2.10	2.20	0.51	3.91	3.11	0.91	1.40	1.00	0.00	2.40	2.00	0.63	2.32	1.90	0.60
Soil and water salinity	4.00	3.40	1.40	7.60	4.10	2.20	5.40	2.91	1.60	8.50	5.70	3.21	7.20	4.20	2.10	7.30	4.40	2.10

*Notes*: Jansen (winding stairs) method performed using the winding stairs sensitivity algorithm (WSSA). Total sensitivity index (TSI) gives total order variance of a driver on the FCM model output—*ceteris paribus*. The three drivers with the maximum TSI percentage for each indicator for each farm type are indicated in bold

*MRAO* marginal farms with rice–aquaculture systems and off-farm income, *MRPAO* marginal farms with rice–pulse–aquaculture systems and off-farm income, *SRPAS* small sharecropping farms with rice–pulse–aquaculture systems, *SRPO* small farms with rice–aquaculture systems and off-farm income

This sensitivity analysis of the relationships between drivers and indicators also showed that farmers perceive that extension, micro-credit, and drainage would reduce fallowed land area and increase their capacity to irrigate both irrigated and rainfed crops. These drivers were also positively related to the FCM concepts for food security and income. There was a strong perceptual agreement among marginal and small farm types on the relationship between sharecropping within polders and increased access to extension, credit availability, and canal dredging. Outside polders, the strongest perceptual linkages were those observed for the SRPAS (Cluster OP-3) typology; within polders, linkages were strongest for the MROA and MPROA (Clusters WP-1 and WP-2) typologies. An increase in canal dredging, however, appears to have stronger linkages with farmers’ perceived capacity to irrigate, regardless of their location within or outside polders.

## Discussion and conclusions

Bangladesh has the highest levels of poverty in South Asia, and about 87 percent of Bangladeshi rural households rely on agriculture for food security and income (Gautam & Faruqee, 2016). A major development objective in Bangladesh is the intensification of farming systems by increasing the number of crops grown on the same unit of land per year. The seventh five-year plan of Bangladesh aligns with the SDGs of the United Nations and is aimed at ending hunger, achieving food security, and improving nutrition through the implementation of more intensive and sustainable agricultural practices. More than USD 7 billion of international donor investment has been requested by the GoB to support the ‘master plan’ for developing the country’s coastal region, with a strong emphasis on reducing land fallowing in the *rabi* season and establishing irrigated double cropping; an estimated USD 500 million has been allocated to this purpose alone (MOA & FAO, 2013).

Such international or national development goals and associated policies—particularly those that pertain to agriculture—often do not fully account for the priorities and perceptual frameworks of rural communities (Aravindakshan et al., 2021). Yet, recent literature underscores the importance of embedding farmer knowledge and perceived impacts of drivers on their farming systems in agricultural policymaking (Tittonell et al., 2016). By integrating farm typological analysis with socio-cognitive modeling in the form of FCM, we studied how farmers in Bangladesh’s coastal farming systems perceive the structure and functioning of their farming systems and how they believe that interventions aimed at facilitating double cropping through surface water irrigation in the dry season could affect agronomic, environmental, and social outcomes. Given the farming systems perceptions of each farmer typology group, represented in the FCM, our findings indicate that farmers perceive that both income and food security could be improved by increasing their access to extension and micro-credit in coastal Bangladesh. The importance of agricultural finance and access to quality advice from extension services in developing countries is widely acknowledged in the non-FCM literature (e.g., Aravindakshan et al., 2021; Vanlauwe et al., 2014), including in Bangladesh (Aravindakshan et al., 2018). These themes also appear in FCM studies; for example, Pacilly et al., 2016 on potato farmers in the Netherlands, and Pathinathan and Peter (2014) and Jayashree et al. (2015) on farmers in India, showed the importance of agricultural extension in the successful crop management. According to the FCM for all farm types across locations in the current study, increasing the availability of extension and micro-credit in the relatively remote coastal region would be perceptually linked to farmer interest in replacing fallowed land with increased cultivation of both rainfed and irrigated crops in the winter *rabi* season, thus indicating the potential for intensification.

Our results point to a perceptual linkage among these farm types, indicating the belief that increased access to extension, credit, and canal dredging would positively affect increased surface water use in *rabi* season. Except for the marginal rice–pulse–aquaculture farm typology with off-farm income located outside polders, each FCM for other farm types indicated a belief that their capacity to irrigate during the dry *rabi* season would benefit most from extension services, credit, and the ability to drain fields for land preparation and planting. Setting aside the perceptual linkages between extension and credit on cropping system intensification, the farm types within polders tended to perceive canal dredging as a key concept and action that would increase their capacity to irrigate. This is in opposition to farm types outside polders who perceived drainage to be more important as a prerequisite for irrigation to support intensified cropping. Identification of this perceptual relationship is an important outcome of the sensitivity analysis that would otherwise not be apparent during FGDs or preliminary visual analysis of FCM. Regardless of farm typology, most farmers indicated that they experience stagnant water or excessive soil moisture at the end of the monsoon season and the start of the subsequent *rabi* season, due to the low-lying fields that prevail in much of the region. As such, field drainage is a likely prerequisite for *rabi* season cropping and the timely establishment of crops such as maize, wheat, and mungbean. Similar results have been observed in farm community participatory agronomic studies to the west of our study area within polders (Yadav et al., 2020).

The FCM analysis indicated that farmers perceive that the rainfed crop area would increase as a function of higher farm-gate prices, though sensitivity analysis suggested that rainfed crop area is highly sensitive to prices, which differs considerably between farm types. Two recent studies (Hossain et al., 2018; Islam et al. 2020) attributed soil salinity as the main factor for low cropping intensity and dry season land fallowing in coastal Bangladesh. Our results surprisingly indicated that for farm types in polders in our study area, increases in soil and water salinity tend to have weak sensitivities for the indicators of cropping system intensification (food security and income outcomes). This may be the result of our sampling location, which, despite being far to the south of Bangladesh’s central coastal area, is slightly north of more saline affected areas. Another surprising result was the low centrality of sharecropping across farm types in both locations, showing the lesser importance of sharecropping arrangements in system intensification, despite the commonality of several kinds of informal sharecropping arrangements in these locations. These results—which are somewhat counterintuitive considering the predominant literature—warrant further behavioral science research on water management and biophysical concepts as a core focus of SI in similar geographies in South Asia. Conflicting approaches (e.g., SI and agroecological intensification) and diverging interests (public vs. private) may present farmers with too many options, which in turn can paralyze decision-making (Schwartz, 2004). As a follow-up study, it would be beneficial to know how conflicting approaches of intensification and diverse interests affect farmer cognitions. Future studies could also focus on gaining a better understanding of farmer preferences for crops disaggregated by various agroecological systems, and/or by farm type as system-specific and farm-type tailored entry points may be needed for development initiatives aimed at cropping system intensification (Aravindakshan et al., 2020). Analysis of farmers’ feedback on the FCMs can help understand how far this research has captured farmer realities, and it can be a part of the follow-up research too. While farmers across farm types both within and outside polders tended to have high centrality values for irrigated and rainfed crops, our study did not elucidate which specific crop species would be both agronomically and socially acceptable in the central coast of Bangladesh. On-farm participatory research that combines agronomic and water management interventions designed to respond to the specific needs of the region’s distinct farm types could be a useful starting point.

The use of FCM to describe farmers’ cognitive frameworks was useful in gaining an improved understanding of the dynamics of farming systems, as perceived by farmers belonging to different farm types, and examining the relative importance of drivers affecting perceptions of crop intensification processes. Though novel methods to assess the efficacy of SI approaches and intensification pathways are currently being tested globally, the use of FCM and similar approaches are of increasing interest, both for *ex ante* planning and targeting research, in addition to *ex post* impact assessments in developing countries. The FCM methodology and many of the results of the current study are likely to be applicable to similar coastal farming systems and deltaic environments in South Asia; for example, those within Bangladesh or parts of eastern India with comparable climates, soils and agricultural practices, demographics, and other socioeconomic factors. In addition, our developed fuzzy cognitive maps are broadly applicable for many farmers in our study area, since we used a survey and averaged scores by farm types. A similar observation was made by Halbrendt et al. (2014), who used a similar survey approach to develop community fuzzy cognitive maps for a large group of Nepalese farmers. Niskanen (2020) studied FCM from a statistical standpoint and showed the analogy between FCMs and linear regression; nonetheless, while FCMs measure strength of relationship between system concepts as perceived by respondent subjects, linear regression estimates the relationship using measured values of factor variables. Future research that investigates these factors comparing participatory FCM with survey results and developing community-wide FCM is warranted.

In consideration of the policies aimed at improving livelihoods in coastal Bangladesh, including those championed by the GoB (e.g., MOA-FAO, 2013), this study demonstrates the value of reflection on the differing perspectives of farmers both within and outside polders to identify entry points for development interventions. In addition, the current study underscores the need for micro-farming systems-level research to assess the context-based feasibility of introduced interventions as perceived by farmers of different farm types. In addition to developing an improved understanding of the complexity of these socioecological systems, the use of FCM and similar approaches could be useful for informing policies to embed the priorities of farming communities in development planning from the bottom-up.

## Supplementary Information

Below is the link to the electronic supplementary material.

Supplementary file1 (DOCX 458 kb)

## Footnotes

1 Farm typologies and the characterization of farming systems have been widely used to understand system complexity and agricultural development trajectories by simplifying and organizing farms into separate but relatively homogenous groups (farm type) internally (Aravindakshan et al., 2020).
