# Supplementary material for: Socio-cognitive constraints and opportunities for sustainable intensification in South Asia: insights from fuzzy cognitive mapping in coastal Bangladesh
Source: Environ Dev Sustain. 2021 Apr 3;23(11):16588–616. doi: 10.1007/s10668-021-01342-y (PMC8550745; doi:10.1007/s10668-021-01342-y)
Supplement: Supplementary file 1 — Supplementary file1 (DOCX 458 kb) [file 10668_2021_1342_MOESM1_ESM.docx]

SUPPLEMENTARY MATERIALS

**Table S1: Descriptive statistics for key structural and functional farm variables used in the analyses, disaggregated for polder and non-polder environments**

| **Variables** | **Unit** | **Farms outside polders (*n=204*)** | |  | **Farms within polders (*n=298*)** | |
| --- | --- | --- | --- | --- | --- | --- |
|  |  | ***Mean*** | ***SD*** |  | ***Mean*** | ***SD*** |
| ***Candidate typology variables*** |  |  |  |  |  |  |
| 1. *Structural variables* |  |  |  |  |  |  |
| Age of the household head | Years | 50.08 | 12.66 |  | 47.14 | 12.55 |
| Education of the household head | Years | 5.44 | 3.60 |  | 4.94 | 3.29 |
| Household size of the household | Numbers | 5.89 | 2.07 |  | 5.65 | 2.19 |
| Total land owned by the household | ha | 0.41 | 0.38 |  | 0.54 | 0.46 |
| Tropical livestock unit^a^ | TLU farm^–1^ | 0.38 | 0.56 |  | 1.05 | 1.41 |
| Pond area under aquaculture | ha | 0.04 | 0.24 |  | 0.08 | 0.22 |
| Sharecropping intensity^b^ | % | 0.31 | 0.30 |  | 0.29 | 0.28 |
| Total family labor used on farm | psd year^–1^ | 163.41 | 106.68 |  | 181.79 | 105.26 |
| Total hired labor used on farm | psd year^–1^ | 75.83 | 47.48 |  | 74.22 | 56.22 |
| Annual income of the household (’000) | BDT | 125.48 | 136.17 |  | 159.01 | 186.62 |
| Annual net savings of the household (’000) | BDT | 20.30 | 26.85 |  | 27.60 | 35.95 |
| 1. *Functional variables* |  |  |  |  |  |  |
| Area under cash crops | ha | 0.37 | 0.33 |  | 0.43 | 0.30 |
| Area under food crops | ha | 0.74 | 0.50 |  | 0.69 | 0.59 |
| Gross cropped area | ha | 1.10 | 0.67 |  | 1.12 | 0.80 |
| Cropping intensity^c^ | % | 168.57 | 18.25 |  | 151.95 | 28.12 |
| Area under irrigation | ha | 0.03 | 0.08 |  | 0.05 | 0.10 |
| Amount of *Aman* season fallow | ha | 0.04 | 0.12 |  | 0.25 | 0.45 |
| Amount of *Rabi* season fallow | ha | 0.39 | 0.33 |  | 0.44 | 0.30 |
| Share of total crops sold | % | 80.34 | 14.54 |  | 85.31 | 8.80 |
| Months of food self-sufficiency | Number of months | 10.30 | 2.18 |  | 10.08 | 2.34 |
| Share of expenditure for food | % | 62.48 | 18.00 |  | 58.88 | 17.96 |
| Off-farm income | % | 56.60 | 27.35 |  | 49.42 | 30.46 |
| Remittances received per annum (‘000) | BDT | 10.66 | 19.76 |  | 14.37 | 23.40 |
| Notes: psd=person-day, which is 8 hours of work; 1USD was ~78 BDT in 2015; ^a^ Tropical livestock unit (TLU) calculated according to method in Harvest Choice, 2015 (https://harvestchoice.org/data/an05_tlu); ^b^ Sharecropping intensity is ratio of sharecropped land to total land available for cultivation in %; ^c^ Cropping intensity averaged across fields calculated as $\frac{Gross harvested area \left( ha \right) {farm}^{-1} {year}^{-1}}{Total land area \left( ha \right) {farm}^{-1} {year}^{-1}}\times100$ and exceeds 100% where double or triple cropping is practiced. All monetary values are nominal. | | | | | | |

**Table S2. Relationships between concepts in the system (outside polders)**

|  | ***MRAO*** | |  | ***MRPAO*** | |  | ***SRPAS*** | |  | ***P*-value** |
| --- | --- | --- | --- | --- | --- | --- | --- | --- | --- | --- |
|  | **Mean** | **SD** |  | **Mean** | **SD** |  | **Mean** | **SD** |  |  |
| Market prices (irrigated crops) – Irrigated crops area | 0.272 | 0.227 |  | 0.261 | 0.178 |  | 0.243 | 0.280 |  | 0.515 |
| Market prices (rainfed crops) – Rainfed crops area | 0.399 | 0.337 |  | 0.405 | 0.244 |  | 0.344 | 0.263 |  | 0.575 |
| Microcredit – Capacity to irrigate | 0.612 | 0.328 |  | 0.520 | 0.353 |  | 0.690 | 0.340 |  | 0.117 |
| Microcredit – Fertilizer access | 0.662 | 0.213 |  | 0.536 | 0.366 |  | 0.598 | 0.411 |  | 0.539 |
| Agricultural extension – Capacity to irrigate | 0.663 | 0.333 |  | 0.592 | 0.351 |  | 0.744 | 0.320 |  | 0.179 |
| Agricultural extension – Irrigated crops area | 0.395 | 0.432 |  | 0.572 | 0.370 |  | 0.804 | 0.222 |  | 0.004*** |
| Agricultural extension – Rainfed crops area | 0.742 | 0.381 |  | 0.568 | 0.324 |  | 0.570 | 0.378 |  | 0.136 |
| Drainage – Irrigated crops area | 0.406 | 0.143 |  | 0.362 | 0.269 |  | 0.277 | 0.123 |  | 0.120 |
| Drainage – Rainfed crops area | 0.587 | 0.388 |  | 0.464 | 0.311 |  | 0.133 | 0.285 |  | 0.000*** |
| Canal dredging – Canal water level | 0.742 | 0.356 |  | 0.847 | 0.246 |  | 0.732 | 0.524 |  | 0.703 |
| Irrigated crops area – Rainfed crops area | –0.280 | 0.416 |  | –0.308 | 0.564 |  | –0.438 | 0.546 |  | 0.466 |
| Irrigated crops area – Household income | 0.349 | 0.115 |  | 0.316 | 0.194 |  | 0.300 | 0.293 |  | 0.797 |
| Irrigated crops area – Food security | 0.256 | 0.096 |  | 0.314 | 0.202 |  | 0.270 | 0.291 |  | 0.374 |
| Irrigated crops area – Fallow | –0.534 | 0.252 |  | –0.495 | 0.293 |  | –0.383 | 0.344 |  | 0.364 |
| Rainfed crops area – Irrigated crops area | –0.280 | 0.416 |  | –0.308 | 0.564 |  | –0.438 | 0.546 |  | 0.466 |
| Rainfed crops area – Household income | 0.544 | 0.107 |  | 0.575 | 0.168 |  | 0.543 | 0.177 |  | 0.647 |
| Rainfed crops area – Food security | 0.400 | 0.284 |  | 0.377 | 0.326 |  | 0.539 | 0.300 |  | 0.140 |
| Rainfed crops area – Fallow | –0.537 | 0.411 |  | –0.448 | 0.280 |  | –0.449 | 0.311 |  | 0.584 |
| Fallow – Irrigated crops area | –0.534 | 0.252 |  | –0.495 | 0.293 |  | –0.343 | 0.329 |  | 0.134 |
| Fallow – Rainfed crops area | –0.127 | 0.163 |  | –0.448 | 0.280 |  | –0.422 | 0.320 |  | 0.002*** |
| Access to fertilizer – Irrigated crops area | 0.213 | 0.149 |  | 0.214 | 0.217 |  | 0.465 | 0.354 |  | 0.008 |
| Access to fertilizer – Rainfed crops area | 0.187 | 0.245 |  | 0.118 | 0.208 |  | 0.353 | 0.266 |  | 0.002*** |
| Hired labor – Irrigated crops area | 0.238 | 0.149 |  | 0.257 | 0.303 |  | 0.451 | 0.347 |  | 0.041* |
| Hired labor – Rainfed crops area | 0.340 | 0.235 |  | 0.347 | 0.311 |  | 0.429 | 0.238 |  | 0.373 |
| Sluice gate control – Canal water level | 0.535 | 0.395 |  | 0.618 | 0.435 |  | 0.773 | 0.402 |  | 0.050* |
| Canal water level – Capacity to irrigate | 0.663 | 0.527 |  | 0.767 | 0.299 |  | 0.570 | 0.365 |  | 0.053* |
| Capacity to irrigate – Sharecropping | 0.228 | 0.392 |  | 0.379 | 0.379 |  | 0.518 | 0.418 |  | 0.123 |
| Sharecropping – Irrigated crops area | 0.305 | 0.211 |  | 0.291 | 0.166 |  | 0.356 | 0.132 |  | 0.275 |
| Sharecropping – Rainfed crops area | 0.406 | 0.143 |  | 0.503 | 0.273 |  | 0.462 | 0.298 |  | 0.099 |
| Household income – Capacity to irrigate | 0.561 | 0.308 |  | 0.496 | 0.294 |  | 0.544 | 0.382 |  | 0.751 |
| Household income – Access to fertilizer | 0.408 | 0.370 |  | 0.387 | 0.376 |  | 0.637 | 0.359 |  | 0.036* |
| Household income – Hired labor | 0.382 | 0.316 |  | 0.545 | 0.367 |  | 0.370 | 0.316 |  | 0.101 |
| Household income – Sluice gate control | 0.178 | 0.287 |  | 0.142 | 0.264 |  | 0.279 | 0.480 |  | 0.299 |
| Household income – Sharecropping | 0.433 | 0.379 |  | 0.513 | 0.396 |  | 0.638 | 0.407 |  | 0.201 |
| Household income – Food security | 0.664 | 0.346 |  | 0.751 | 0.281 |  | 0.596 | 0.333 |  | 0.124 |
| Notes: MRAO=marginal rice–aquaculture systems with off-farm income; MRPAO=marginal rice–pulse–aquaculture systems with off-farm income; SRPAS=small rice–pulse–aquaculture systems with sharecropping; SRPO=small rice–pulse systems with off-farm income; Mean=mean values of relationship weights; SD=standard deviation. Differences between fuzzy cognitive maps assessed by Kruskal–Wallis H test. *, **, and *** indicate significance at the 10%, 5%, and 1% levels, respectively. | | | | | | | | | | |

**Table S3. Relationships between concepts in the system (within polders)**

|  | ***MRPAO*** | |  | ***SRPO*** | |  | ***SRPAS*** | |  | ***P-value*** |
| --- | --- | --- | --- | --- | --- | --- | --- | --- | --- | --- |
|  | **Mean** | **SD** |  | **Mean** | **SD** |  | **Mean** | **SD** |  |  |
| Market prices (irrigated crops) – Irrigated crops area | 0.253 | 0.332 |  | 0.509 | 0.317 |  | 0.295 | 0.325 |  | 0.000*** |
| Market prices (rainfed crops) – Rainfed crops area | 0.269 | 0.274 |  | 0.509 | 0.310 |  | 0.398 | 0.311 |  | 0.009** |
| Microcredit – Capacity to irrigate | 0.783 | 0.250 |  | 0.755 | 0.386 |  | 0.752 | 0.287 |  | 0.659 |
| Microcredit – Fertilizer access | 0.761 | 0.422 |  | 0.860 | 0.247 |  | 0.735 | 0.355 |  | 0.116 |
| Agricultural extension – Capacity to irrigate | 0.880 | 0.250 |  | 0.691 | 0.374 |  | 0.705 | 0.334 |  | 0.106 |
| Agricultural extension – Irrigated crops area | 0.903 | 0.172 |  | 0.748 | 0.277 |  | 0.769 | 0.217 |  | 0.103 |
| Agricultural extension – Rainfed crops area | 0.689 | 0.423 |  | 0.529 | 0.391 |  | 0.559 | 0.399 |  | 0.234 |
| Salinity – Irrigated crops area | –0.269 | 0.306 |  | –0.097 | 0.455 |  | –0.144 | 0.375 |  | 0.298 |
| Salinity – Rainfed crops area | –0.316 | 0.298 |  | –0.156 | 0.456 |  | –0.139 | 0.371 |  | 0.355 |
| Salinity – Capacity to irrigate | –0.166 | 0.312 |  | –0.134 | 0.380 |  | –0.024 | 0.306 |  | 0.076 |
| Drainage – Irrigated crops area | 0.401 | 0.141 |  | 0.331 | 0.270 |  | 0.301 | 0.094 |  | 0.156 |
| Drainage – Rainfed crops area | 0.592 | 0.374 |  | 0.490 | 0.304 |  | 0.122 | 0.263 |  | 0.000*** |
| Canal dredging – Canal water level | 0.854 | 0.175 |  | 0.751 | 0.365 |  | 0.412 | 0.327 |  | 0.000*** |
| Irrigated crops area – Rainfed crops area | –0.260 | 0.489 |  | –0.365 | 0.504 |  | –0.454 | 0.562 |  | 0.092 |
| Irrigated crops area – Household income | 0.370 | 0.332 |  | 0.384 | 0.276 |  | 0.467 | 0.295 |  | 0.158 |
| Irrigated crops area – Food security | 0.497 | 0.321 |  | 0.511 | 0.287 |  | 0.372 | 0.314 |  | 0.016* |
| Irrigated crops area – Fallow | –0.495 | 0.251 |  | –0.364 | 0.261 |  | –0.279 | 0.306 |  | 0.002** |
| Rainfed crops area – Irrigated crops area | –0.260 | 0.489 |  | –0.365 | 0.504 |  | –0.454 | 0.562 |  | 0.092 |
| Rainfed crops area – Household income | 0.656 | 0.278 |  | 0.691 | 0.186 |  | 0.682 | 0.182 |  | 0.930 |
| Rainfed crops area – Food security | 0.704 | 0.245 |  | 0.547 | 0.275 |  | 0.605 | 0.286 |  | 0.039* |
| Rainfed crops area – Fallow | –0.689 | 0.381 |  | –0.360 | 0.286 |  | –0.477 | 0.372 |  | 0.004** |
| Fallow – Irrigated crops area | –0.141 | 0.169 |  | –0.289 | 0.302 |  | –0.319 | 0.251 |  | 0.102 |
| Fallow – Rainfed crops area | –0.519 | 0.282 |  | –0.282 | 0.305 |  | –0.395 | 0.248 |  | 0.005** |
| Access to fertilizer – Irrigated crops area | 0.602 | 0.374 |  | 0.508 | 0.279 |  | 0.390 | 0.352 |  | 0.023* |
| Access to fertilizer – Rainfed crops area | 0.269 | 0.237 |  | 0.390 | 0.238 |  | 0.302 | 0.248 |  | 0.064 |
| Hired labor – Irrigated crops area | 0.560 | 0.312 |  | 0.326 | 0.290 |  | 0.402 | 0.358 |  | 0.031* |
| Hired labor – Rainfed crops area | 0.435 | 0.228 |  | 0.452 | 0.245 |  | 0.415 | 0.276 |  | 0.700 |
| Sluice gate control – Canal water level | 0.760 | 0.357 |  | 0.617 | 0.493 |  | 0.782 | 0.354 |  | 0.112 |
| Canal water level – Capacity to irrigate | 0.734 | 0.268 |  | 0.679 | 0.369 |  | 0.636 | 0.415 |  | 0.957 |
| Capacity to irrigate – Sharecropping | 0.617 | 0.431 |  | 0.454 | 0.408 |  | 0.472 | 0.426 |  | 0.359 |
| Sharecropping – Irrigated crops area | 0.306 | 0.203 |  | 0.293 | 0.161 |  | 0.266 | 0.158 |  | 0.497 |
| Sharecropping – Rainfed crops area | 0.401 | 0.141 |  | 0.489 | 0.286 |  | 0.337 | 0.311 |  | 0.001** |
| Household income – Capacity to irrigate | 0.237 | 0.379 |  | 0.394 | 0.320 |  | 0.633 | 0.304 |  | 0.000*** |
| Household income – Access to fertilizer | 0.640 | 0.380 |  | 0.537 | 0.427 |  | 0.442 | 0.404 |  | 0.191 |
| Household income – Hired labor | 0.713 | 0.411 |  | 0.476 | 0.361 |  | 0.389 | 0.360 |  | 0.006** |
| Household income – Sluice gate control | 0.142 | 0.312 |  | 0.298 | 0.350 |  | 0.489 | 0.393 |  | 0.001** |
| Household income – Sharecropping | 0.309 | 0.442 |  | 0.458 | 0.390 |  | 0.553 | 0.409 |  | 0.104 |
| Household income – Food security | 0.640 | 0.307 |  | 0.626 | 0.360 |  | 0.670 | 0.348 |  | 0.682 |
| Notes: MRPAO=marginal rice–pulse–aquaculture systems with off-farm income; SRPAS=small rice–pulse–aquaculture systems with sharecropping; SRPO=small rice–pulse systems with off-farm income; Mean=mean values of relationship weights; SD=standard deviation. Differences between fuzzy cognitive maps assessed by Kruskal–Wallis H test. *, **, and *** indicate significance at the 10%, 5%, and 1% levels, respectively. | | | | | | | | | | |

| **Box S1** |
| --- |
| FCMs are graphical representations of the knowledge/mental constructs of individuals’ perception of a given system (e.g. farming system). Any FCM consists of four elements: (1) system components (concepts / nodes), (2) directed lines connecting each of the system concepts, (3) values or weights assigned by individuals to measure the strength of relationship between concepts (4) driver variables represented by self-looped arrows. These four elements represent the mapped system of the mental model. The directed lines labeled with fuzzy values show the strength of the causal conditions between the concepts/factors. A fuzzy cognitive map is a model of system structure as perceived by the respondent farmers.  In this study, FCMs are constructed using the ‘FuzzyDANCES’ software developed by Wageningen University and Research, the Netherlands. FuzzyDANCES can be freely downloaded from here : [https://fse.models.gitlab.io/COMPASS/FuzzyDANCES/](https://eur03.safelinks.protection.outlook.com/?url=https%3A%2F%2Ffse.models.gitlab.io%2FCOMPASS%2FFuzzyDANCES%2F&data=04%7C01%7Csreejith.aravindakshan%40wur.nl%7Cc60d07f4311f4ced8ccf08d8cc702d13%7C27d137e5761f4dc1af88d26430abb18f%7C0%7C0%7C637484129469812902%7CUnknown%7CTWFpbGZsb3d8eyJWIjoiMC4wLjAwMDAiLCJQIjoiV2luMzIiLCJBTiI6Ik1haWwiLCJXVCI6Mn0%3D%7C1000&sdata=jjcuUKvfFGo45mAgt8fnIsAQ8RKu%2FPxulat86TDDOIU%3D&reserved=0)  The FuzzyDANCES comprises three windows: the main **data window** (left, top) is where, **the map window** (right) to draw the FCM and **the plot window** (left, bottom) where the results of the iterative matrix multiplications are shown (Figure S1).    **Figure S1.** Graphical user interface of FuzzyDANCES  In the map window, concepts are created by creating boxes with unique colors each for a given concept. The direction of influence/relationship lines from a given concept (cause) to another concept (effect) is also given the same color as the concept that causes the influence. In the main data window, the average weights assigned by respondent farmers based on a 7-point Likert scale, signifying the positive or negative influence and degree of strength for each causal relationship are entered between the relationship lines. |

| **Box S2** |
| --- |
| ***Farm types identified outside polder area^^[[1]](#footnote-1)^^***  A detailed characterization of farm types is provided below:  **Cluster OP-1.** *Marginal farms with rice–aquaculture systems and off-farm income (MRAO):*  This distinct group of marginal farms comprised 15% of the sample. The average landholding for this group was around 0.2 ha. They grow *aman* rice in the *kharif* season. In the dry *rabi* season, vegetables are grown by households with irrigation facilities, while about 20–25% of farmland is left fallow. Apart from cropping, farmers derive supplementary income from pond aquaculture of *Tilapia* and *Pangasius* spp. Cropping provides only 30% of household income, with 60–65% of household income derived from off-farm income, such as rickshaw pulling or working as hired labor. Households comprise 5–7 members, who contribute 65% of the total farm labor. The average annual rate of nitrogen applied is 65 kg ha^–1^.  **Cluster OP-2**. *Marginal farms with rice–pulse–aquaculture systems and off-farm income (MRPAO):* This group was the major farm type outside polders, accounting for 60% of the sample. On average, farmers own 0.33 ha of land, nearly 100% of which is cultivated under *aman* rice in the *kharif* monsoon season. In the *rabi* season, farmers devote ~45–50% of their land for pulse cultivation (mungbean or lathyrus (grass pea)), leaving the remainder in fallow. While 95% of the pulse crop is sold, only 10–15% of the rice is sold. Household members are also engaged in off-farm income-generating activities, such as rickshaw pulling, shop management, or construction work. About 70% of their income is derived off-farm. They also maintain pond aquaculture within the homestead, on about 0.06 ha, mainly for home consumption. Households have 5–6 members, with 76% of farm labor supplied by the household. Urea is the main fertilizer used, at the rate of 65–70 kg N ha^–1^ year^–1^.  **Cluster OP-3.** *Small sharecropping farms with rice–pulse–aquaculture systems (SRPAS):*  This group of small farms (25% of sample) is primarily engaged in sharecropping of rice and pulses. They own 0.7 ha of land on average. In the *kharif* monsoon season, nearly 100% of the land is under rice, while in the dry season (*rabi*), 30% is under mungbean, 20% is for other pulses, such as lentil and grass pea, and the remainder is in fallow. Some parts of the cultivated land during *rabi* may be under *boro* rice on farms with access to irrigation. Otherwise, mungbean is grown. Pond aquaculture is common, on about 0.06 ha. The fish serve as a protein source for the household. On average, there are six members per household, with 50–60% of the total labor contributed by family labor. The rate of nitrogen application to crops is 85–90 kg ha^–1^ year^–1^, which is remarkably higher than the other farm types outside polders.  ***4.1.2 Farm types identified within polder areas***  **Cluster WP-1**. *Marginal farms with rice–pulse–aquaculture systems and off-farm income (MRPAO):* Of the farm households sampled within polder areas, 25% belong to this farm type. The average land owned is 0.21 ha. During *kharif*, rice is cultivated on the entire farm area*,* with 50% left fallow in the subsequent *rabi* season and the remainder used to cultivate mungbean and groundnut. Apart from cropping, farmers are engaged in pond aquaculture on 0.05 ha. Crucially, the major source of household income (82%) comes from off-farm, such as rickshaw pulling and seasonal labor migration to Dhaka. On average, households have 4–6 members, with more than 85% of the labor contributed by the household. Annual nitrogen use on the farms is 68–70 kg ha^–1^.  **Cluster WP-2.** *Small farms with rice–aquaculture systems and off-farm income (SRPO):*  This group of small farms own 0.46 ha of land, on average, and represents 49% of the sample. All farmland is cultivated under rice during *kharif*, with 95% of the land in fallow in the *rabi* season. Pond aquaculture occupies 0.04 ha of the homestead area, where local fish species are grown alongside tilapia and grass carp. About 42% of household income comes from off-farm, such as petty shop keeping. Households comprise 4–5 family members, with at least one member working as wage labor outside the village and more than 75% of the labor for farming contributed by the family. Fertilizer use is comparatively low: nitrogen rates average approximately 46 kg ha^–1^ year^–1^.  **Cluster WP-3.** *Small sharecropping farms with rice–pulse–aquaculture systems (SRPAS):*  This farm type is the second largest group within polders, representing 26% of the farms sampled. They are sharecroppers with roughly 0.9–1.0 ha of land that is securely titled with tenure arrangements. Since farming is their main livelihood source, rice is grown on both own and rented land during *kharif*. Almost 45–50% of farmland is left fallow in the winter *rabi* season, with the remainder devoted to rainfed mungbean. Mungbean is grown as a cash crop, with 98% sold on the market. The farms also have ponds within their homesteads for fish culture, with the catch mainly used for home consumption. Households have 5–6 members, with ~58% of the farm labor coming from the family. Urea is the main fertilizer applied at a rate of 78–80 kg N ha^–1^ year^–1^. |


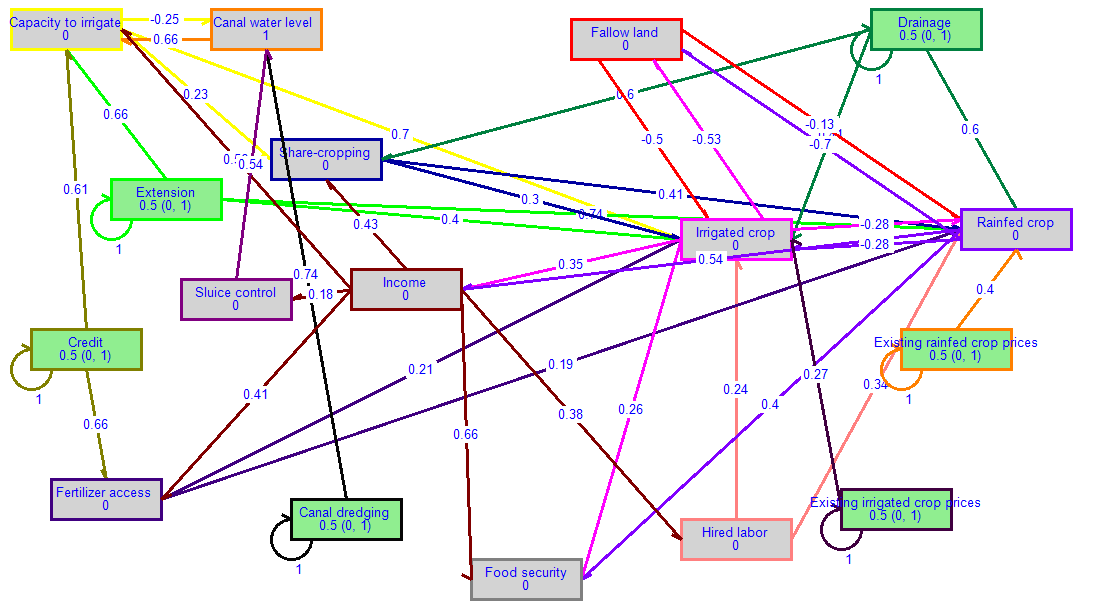


**Figure S2.** FCM output from FuzzyDANCES for the farm type MRAO outside polders


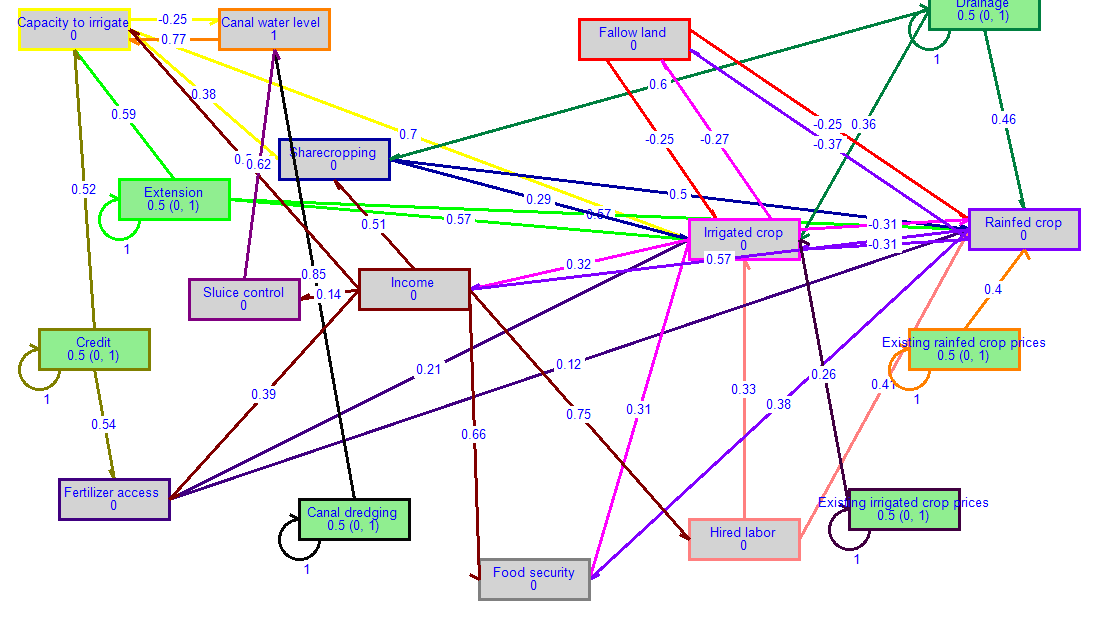


**Figure S3.** FCM output from FuzzyDANCES for the farm type MRPAO outside polders


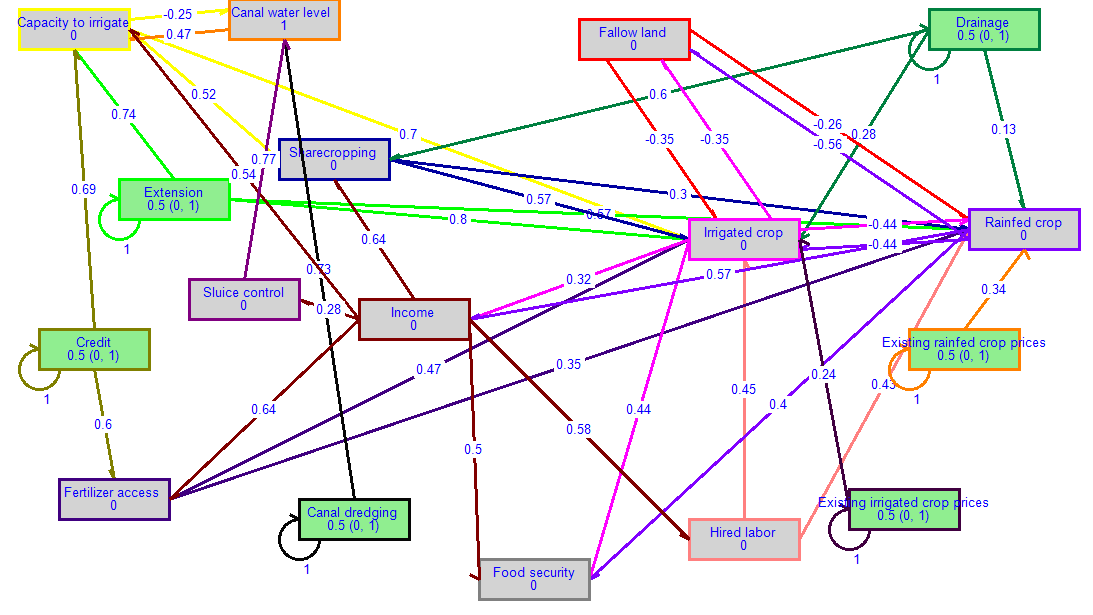


**Figure S4.** FCM output from FuzzyDANCES for the farm type SRPAS outside polders


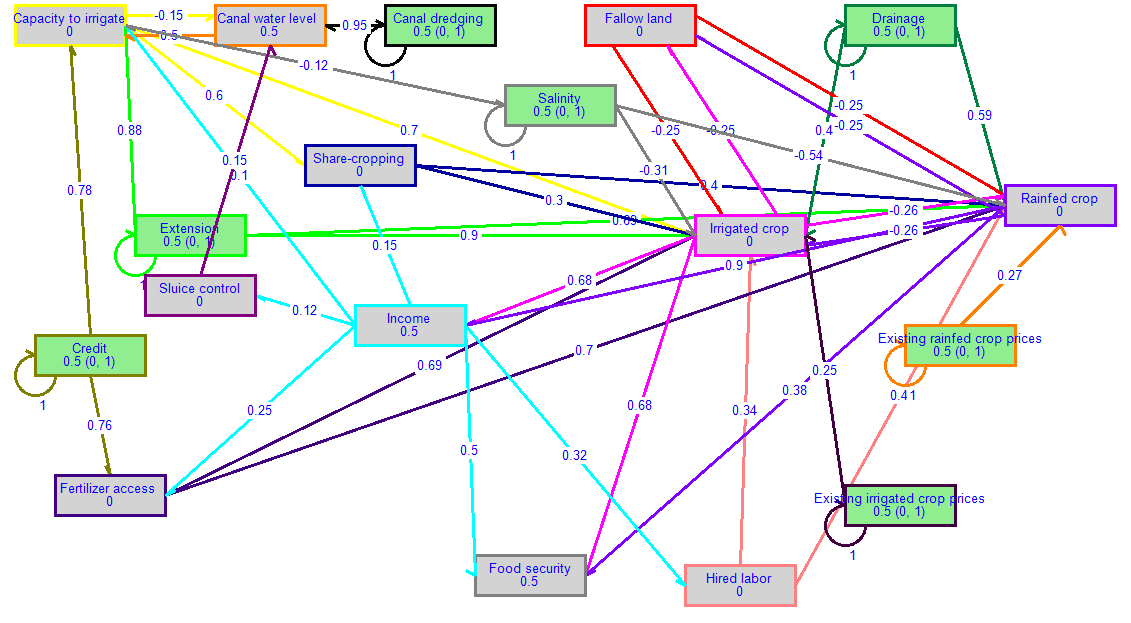


**Figure S5.** FCM output from FuzzyDANCES for the farm type MRPAO within polders


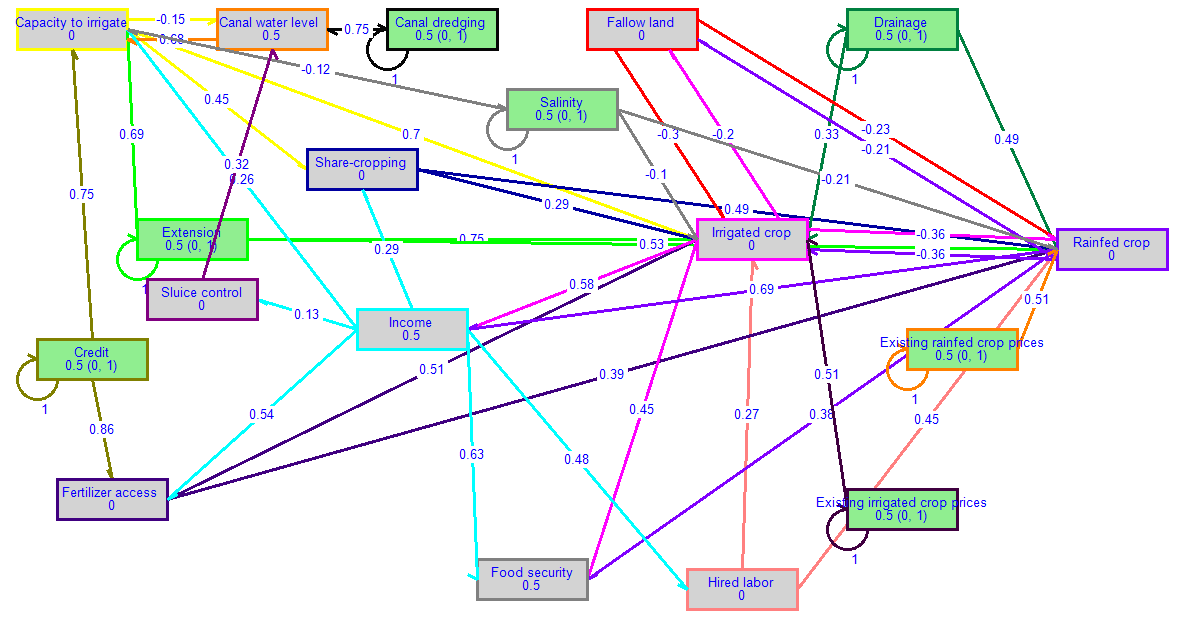


**Figure S6.** FCM output from FuzzyDANCES for the farm type SRPO within polders


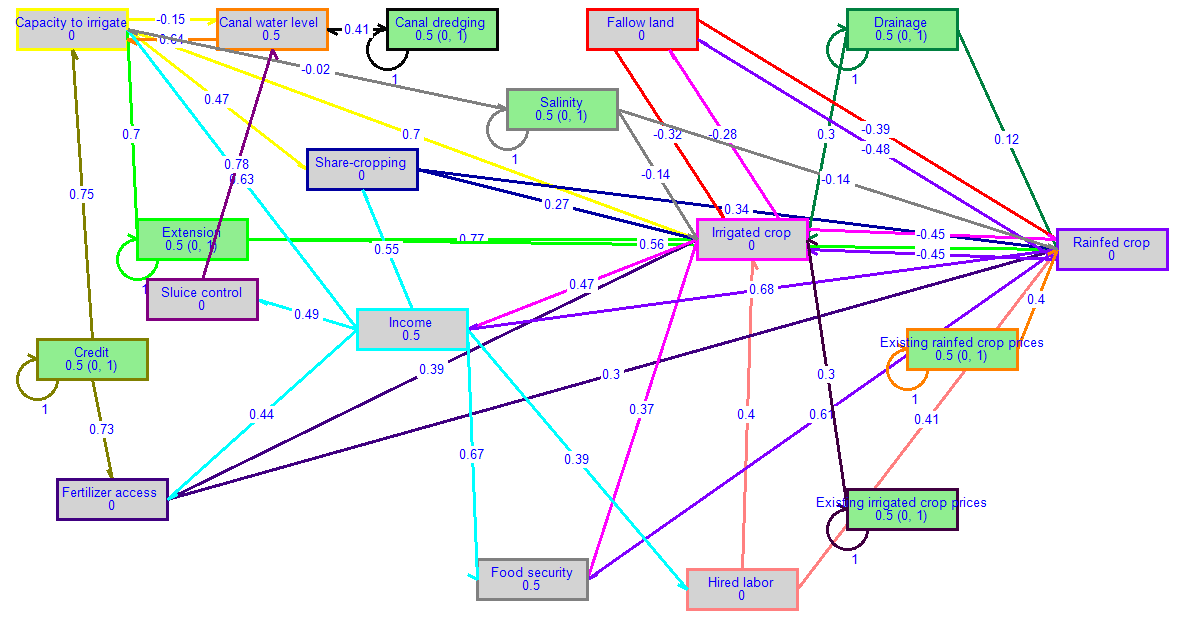


**Figure S7.** FCM output from FuzzyDANCES for the farm type SRPAS within polders

1. The "Marginal farms with rice-pulse-aquaculture systems and off-farm income (MRPAO)" as well as Small sharecropping farms with rice–pulse–aquaculture systems (SRPAS)” both within and outside the polder environments are similar in functions and farm enterprises and households’ structural components.” [↑](#footnote-ref-1)
